# Repurposing of colchicine: critical examination of clinical trials and scoping review

**DOI:** 10.1007/s00228-026-04149-w

**Published:** 2026-08-20

**Authors:** Lilly Josephine Bindel, Roland Seifert

**Affiliations:** Institute of Pharmacology, Hannover Medical School, 30625 Hannover, Germany

**Keywords:** Colchicine, Repurposing, Clinical trials, COVID-19, Cardiovascular disease, Anti-inflammatory, Adverse effect

## Abstract

**Purpose:**

Drug repurposing represents a cost-effective and time-efficient strategy to expand therapeutic treatment options. Colchicine, traditionally used for gout and familial Mediterranean fever, has gained attention as a candidate for diverse conditions. This study aimed to provide a comprehensive and critical overview of clinical trials on colchicine repurposing, with particular focus on reliability, significance, and safety outcomes.

**Methods:**

A structured search of clinicaltrials.gov and the World Health Organization (WHO) International Clinical Trials Registry Platform (ICTRP) was completed on July 07 2026. Following the Preferred Reporting Items for Systematic reviews and Meta-Analyses extension for Scoping Reviews (PRISMA-ScR) guideline, PubMed and Scopus were searched using the term “colchicine AND repurposing”.

**Results:**

40 clinical trials were included. Nine addressed COVID-19, 26 cardiovascular conditions or related interventions, and five with other indications. Evidence in COVID-19 was inconsistent. Cardiovascular evidence was strong for chronic coronary artery disease and prevention after myocardial infarction. Findings in acute coronary syndromes, percutaneous coronary intervention, surgery and heart failure were mixed and often limited to surrogate endpoints. Other indications did not observe a significant benefit. Most frequent adverse effects were gastrointestinal, particularly diarrhoea. Increased non-cardiovascular mortality in individual trials highlights toxicity potential.

**Conclusion:**

Current evidence most consistently supports colchicine for secondary cardiovascular prevention in established coronary disease. No reliable evidence is found for benefit across broader cardiovascular conditions, COVID-19 or other indications. A narrow therapeutic index and high drug-interaction potential necessitate careful patient selection and monitoring. Well-powered and methodologically robust clinical trials are required to investigate and confirm potential benefits.

**Supplementary information:**

The online version contains supplementary material available at https://doi.org/10.1007/s00228-026-04149-w.

## Introduction

The need for beneficial treatments to reduce the burden of disease is important [27], but the development of new drugs is expensive and time-consuming [121]. Another principle for the expansion of treatment options is the repurposing of already approved and established drugs beyond their traditional indications [64]. Such a candidate is colchicine, an ancient drug traditionally used in gout and familial Mediterranean fever, while being currently investigated for a variety of other conditions [26, 75]. Its anti-inflammatory and immunosuppressive effect is based on the inhibition of microtubule polymerization and suppression of the release of several proinflammatory cytokines [44]. However, it is characterized by a narrow therapeutic range and high drug-interaction potential [48].

The purpose of the study is to provide a state-of-the-art overview for colchicine of the currently investigated medical conditions for colchicine and the evidence for efficacy and improved outcomes by combining trial registry and literature search. Other available publications about the repurposing of colchicine often focus on single conditions [13, 71] or lack comprehensiveness in the assessment of presented results [4, 93]. Our aim is to provide a comprehensive and balanced overview of evidence for treatment benefits with colchicine, but also a critical examination of findings and study design, as well as adverse effects and toxicity concerns. This will inform and support clinical research and practice.

## Methods

### Selection of clinical trials

The search for clinical trials was conducted in clinicaltrials.gov, an online database of the United States National Library of Medicine (NLM), and the World Health Organization (WHO) International Clinical Trials Registry Platform (ICTRP), with separate searches of the Chinese Clinical Trial Registry (ChiCTR), the Iranian Registry of Clinical Trials (IRCT), the UK’s Clinical Study Registry (ISRCTN), and the Australian New Zealand Clinical Trials Registry (ANZCTR) [6, 20, 20, 92, 142]. The drug analysed was colchicine, with the focus on its repurposing. The search term used in all the clinical trial databases was “colchicine”, and the search was completed on July 07 2026.

No restrictions regarding study year, language, registry location, or study design were applied at the search stage, randomized and non-randomized interventional studies and observational human studies were eligible. Inclusion required listing on an official clinical trial registry, a peer-reviewed publication with extractable results, and the study subject being repurposing of colchicine. This excluded trials without published results, pending clinical trials, studies about the classical indication (gout), studies on bioavailability or pharmacokinetics, and studies where colchicine was not the primary subject. Adverse events were defined according to the Good Clinical Practice (GCP) definition [36, 79]. The analysis was not preregistered.

In two clinical trial databases, it was possible to filter for trials with results, including clinicaltrials.gov (*n* = 48) and the ISRCTN (*n* = 20). In the ANZCTR, it was possible to filter by recruitment status. Trials labelled as “completed”, “withdrawn”, “suspended”, and “stopped early” were included for further screening, resulting in 15 records examined for inclusion. For the ChiCTR and IRCT, it was not possible to filter the clinical trials appropriately. Therefore, both databases were searched manually for the term “colchicine”, resulting in an initial number of 26 records for the ChiCTR and 87 for the IRCT.

### Systematic literature search

The current investigation was conducted following the Preferred Reporting Items for Systematic reviews and Meta-Analyses extension for Scoping Reviews (PRISMA-ScR) [129] (Table S1). A literature search was performed on PubMed (https://pubmed.ncbi.nlm.nih.gov/) and Scopus (https://www.scopus.com/pages/home) on July 08 2026. The completed PubMed strategy was “(“colchicine” [All Fields]) AND (“repurposing” [All Fields])”, without filters or limits. In Scopus, the same terms were searched in title, abstract and keywords. No date or language filters were applied. Eligible sources included peer-reviewed human clinical studies, systematic reviews, meta-analyses and narrative reviews addressing colchicine repurposing. Exclusion criteria were publications on the classical indication (gout), studies on bioavailability or pharmacokinetics, animals or basic drug research, and studies where colchicine was not the primary subject. The number of records for the search term was 89 in PubMed and 156 in Scopus. 56 duplicate listings were removed with RefWorks. The review was not preregistered.

## Results

### Overview of included clinical trials

**Fig. 1:**
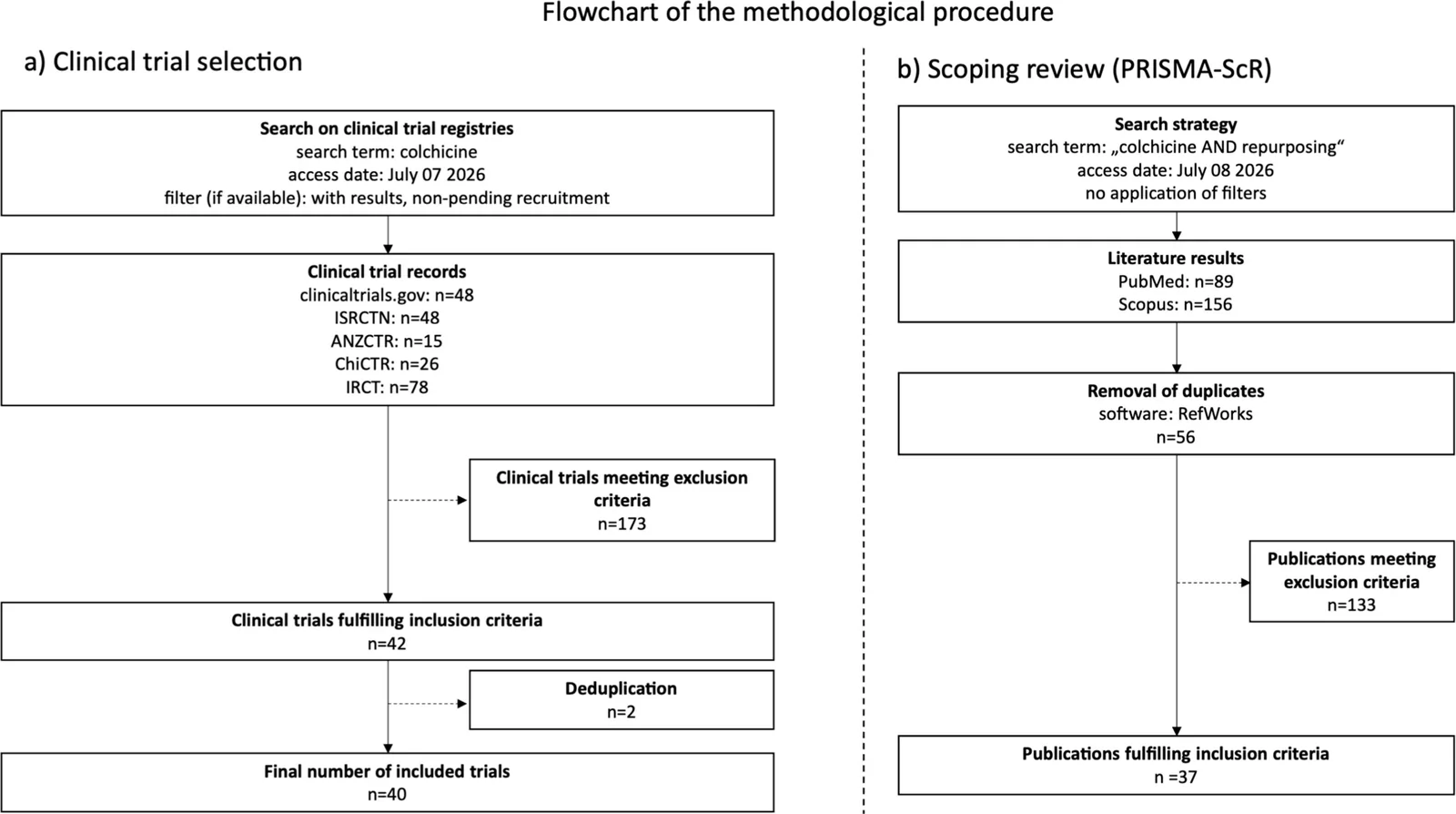
Methodological procedure for **a**) the selection of clinical trials and **b**) the literature search of the scoping review

Figure 1 provides a flowchart of the methodology. The initial search for “colchicine” on clinicaltrials.gov yielded 264 results. Applying restrictions to include only trials with posted results and an available publication reduced this number to 48 (18.2%). These restrictions ensured transparency, completeness, and consistency between sources, minimizing selective reporting and publication bias while improving the reliability of extracted data [1, 12, 84]. The number decreased further when limiting to trials investigating colchicine repurposing, leaving 14 analysed trials (5.3%). Similarly, for the ISRCTN, 31 clinical trials were listed overall, *n* = 20 (66.7%) with results, and *n* = 3 (9.7%) with peer-reviewed results for the repurposing of colchicine that were included. In the ChiCTR, from 26 screened records, 3 (11.5%) clinical trials with published results about repurposing of colchicine were included, similarly for the IRCT with 87 records and 16 inclusions (18.4%), and the ANZCTR with 15 records and 7 trial inclusions (46.7%). After deduplication of records registered in more than one registry, 40 clinical trials were included.

Repurposing topics primarily concerned COVID-19 infections (9 trials) as well as cardiovascular conditions and related interventional procedures (26 trials). Five further trials examined obesity, advanced hepatocellular carcinoma (HCC), actinic keratosis, aphthous stomatitis, and periodic fever, aphthous stomatitis, pharyngitis, and adenitis (PFAPA) syndrome.

**Fig. 2:**
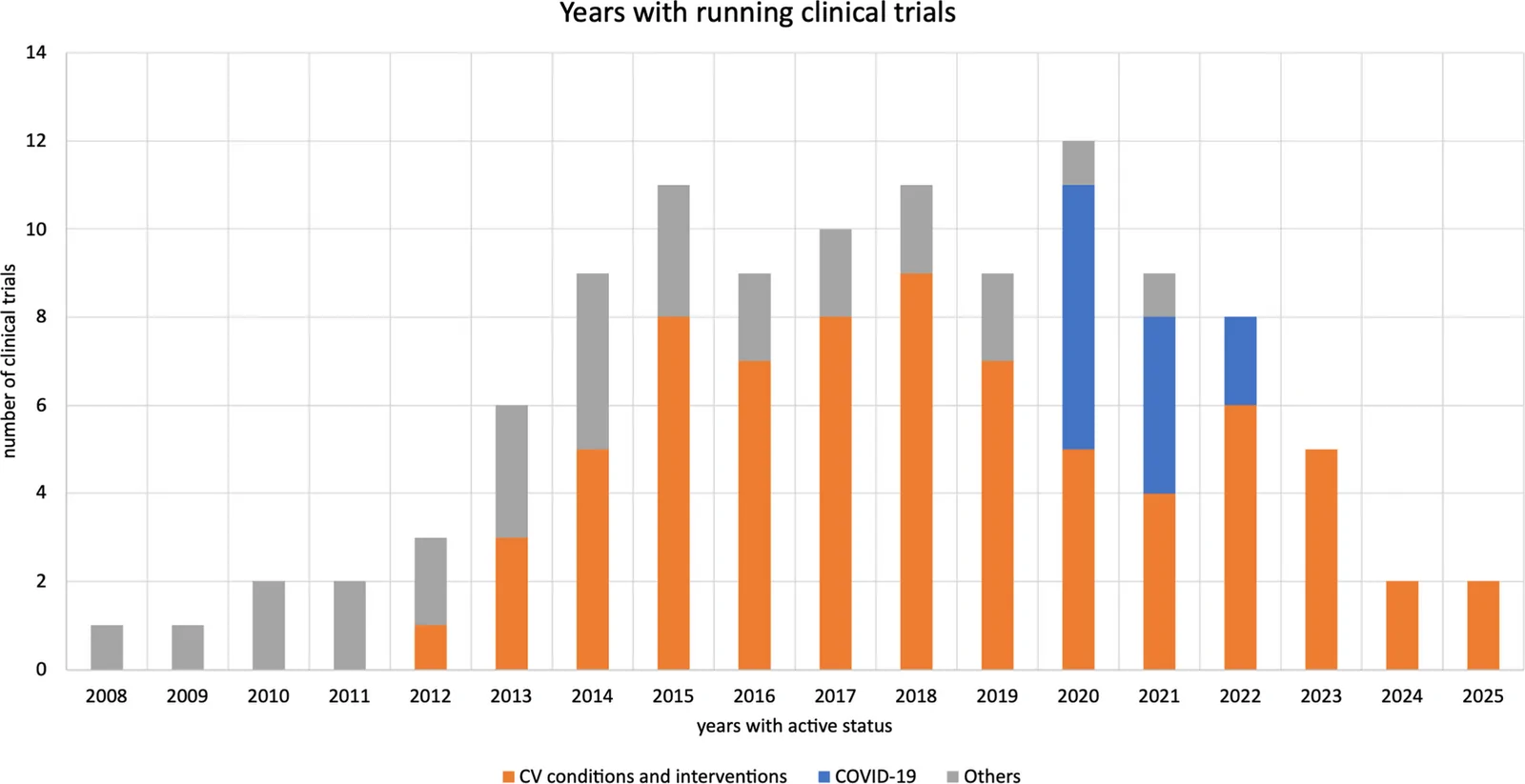
Years with active clinical trials, categorized by repurposing subject

Regarding the time period of trial conduction, most analysed trials started from 2015 onwards, with a peak in 2020 (Fig. 2). Clinical trials about cardiovascular conditions, as well as other smaller repurposing topics, were investigated throughout the whole time period, while studies about COVID-19 emerged in 2020 due to the global pandemic. The lower number in more recent years mainly reflects the interval required to complete trials and prepare publications, because recently completed or ongoing trials are unlikely to have available results.

Study populations included both sexes and predominantly adults (> 18 years) (Table 1 and S2). Exceptions were seen in the Fontan patient trial [45], which included children (20 months to 5 years) due to early surgical timing [17], as well as the investigation of PFAPA syndrome, including children from one to 10 years [49]. Five clinical trials enrolled only older adults, with minimum ages ranging from 35 to 55 years.

**Table 1:**
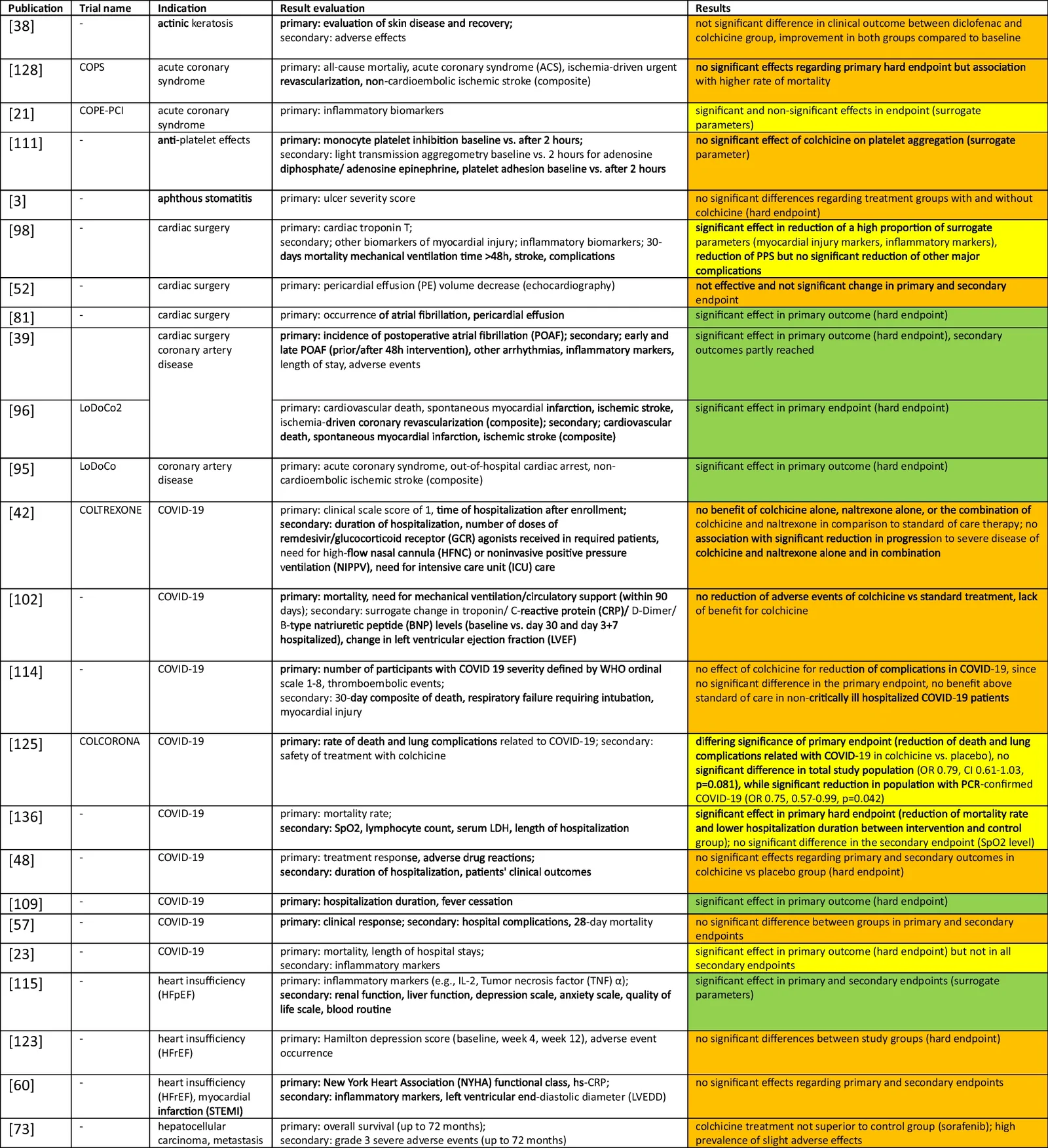

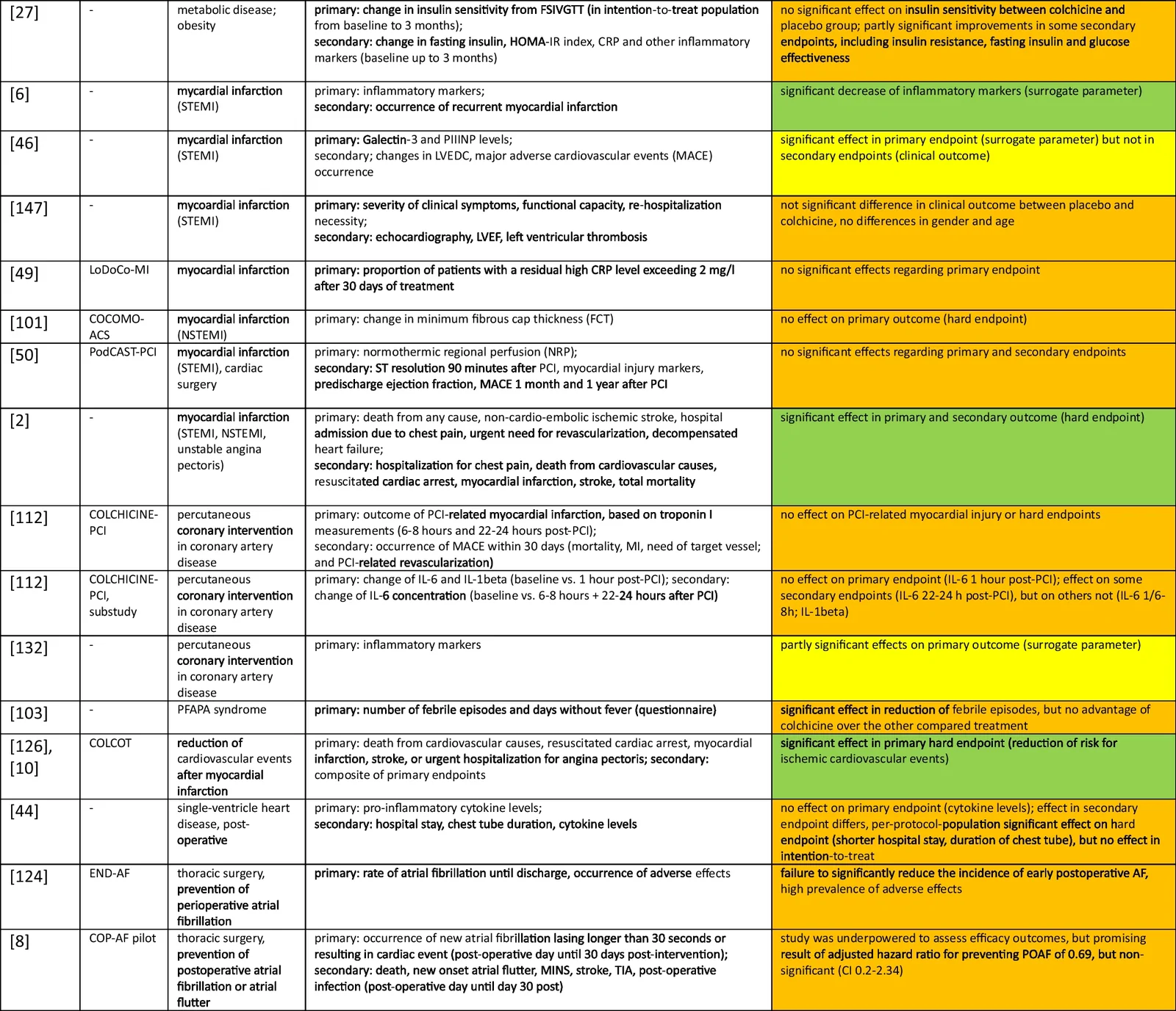
Execution and results of evaluated clinical trials on colchicine repurposing. Information is provided on the cited publication, trial subject outcome evaluation, and results. Cells for outcomes are highlighted in different colours to indicate whether endpoints were significant (*p* < 0.05 vs. *p* > 0.05) and achieved (positive outcome vs. no benefit): green highlights trials where all endpoints were significantly achieved, yellow highlights trials where some endpoints were achieved while others were not, and orange highlights trials where no endpoints were significantly achieved. Further details can be found in Table S2

Inclusion criteria reflected the repurposing focus. COVID-19 trials required a confirmed acute COVID-19 infection, while cardiovascular trials specified relevant conditions such as single-ventricle heart disease, coronary artery disease (CAD), heart failure, or myocardial infarction (MI). Interventional trials additionally required recent procedures such as Fontan surgery, percutaneous coronary intervention (PCI), open-heart surgery, or lung resection. Trials in other repurposing areas required obesity, advanced-stage HCC, PFAPA syndrome, aphthous stomatitis, or actinic keratosis.

### Repurposing areas of colchicine

The use of colchicine has been investigated in various settings. The included clinical trials address acute infections with COVID-19, cardiovascular diseases, thoracic interventions, autoinflammatory conditions, metabolic disorders and advanced HCC. The main results are presented in the following section and in Table 1, while trial characteristics are summarized in Table S2. A detailed overview of each trial can be found in the supplemental results.

In COVID-19 infections, nine trials assessed colchicine alone or as an add-on therapy to standard of care, with contradictory findings. The largest of these, COLCORONA [125], did not significantly reduce death or pulmonary complications in the overall population, although a benefit was observed in polymerase chain reaction (PCR)-confirmed cases. Five trials investigating colchicine alone or in combination with other drugs (e.g. rosuvastatin, naltrexone) failed to significantly improve their primary outcomes [43, 49, 58, 102, 113]. In contrast, three smaller studies reported lower mortality, shorter hospitalisation, or shorter persistence of fever [24, 108, 125, 136].

Twenty-five clinical trials and one mechanistic study were conducted in cardiovascular and interventional settings. Six trials reported a significant benefit in primary hard clinical endpoints [2, 40, 82, 95, 96, 126], while eleven trials failed to prove their primary hard endpoint (a significantly improved clinical outcome) [9, 50, 51, 53, 61, 101, 111, 123, 124, 128, 147]. In myocardial infarction, heart failure and interventional settings, findings were mixed. Most studies did not find improved clinical endpoints, despite effects on some inflammatory markers. Importantly, the study COPS [128] did even observe a significantly negative effect on total and non-cardiovascular death. Regarding surrogate parameters, six studies found a significant or partly significant effect, for example on inflammatory or myocardial injury markers [7, 22, 47, 98, 114, 132], while two trials did not observe a significant difference [45, 111]. Small studies in paediatric cardiac surgery [45] and healthy adults [110] failed to demonstrate consistent anti-inflammatory or antiplatelet effects.

Furthermore, five clinical trials investigated the effects of colchicine on obesity, hepatocellular carcinoma, aphthous stomatitis, actinic keratosis, and PFAPA syndrome [3, 28, 39, 74, 103]. None established a clear comparative clinical benefit. However, when two treatment groups were compared, often both resulted in a significant improvement of the examined condition, for example in PFAPA syndrome, actinic keratosis, and aphthous stomatitis.

## Discussion

### Reliability of analysed repurposing trials

Beyond the results of a clinical trial, it is essential to understand the methodology and the conditions under which the study was performed. These factors strongly influence the reliability and generalizability of the findings. Certain methodological properties can prevent or introduce bias, thereby shaping the interpretation of results. In the following and in Table 2, the key aspects of the analysed repurposing trials are presented and discussed. A detailed overview of these aspects and their relevance is provided in Table S3.

**Table 2:**
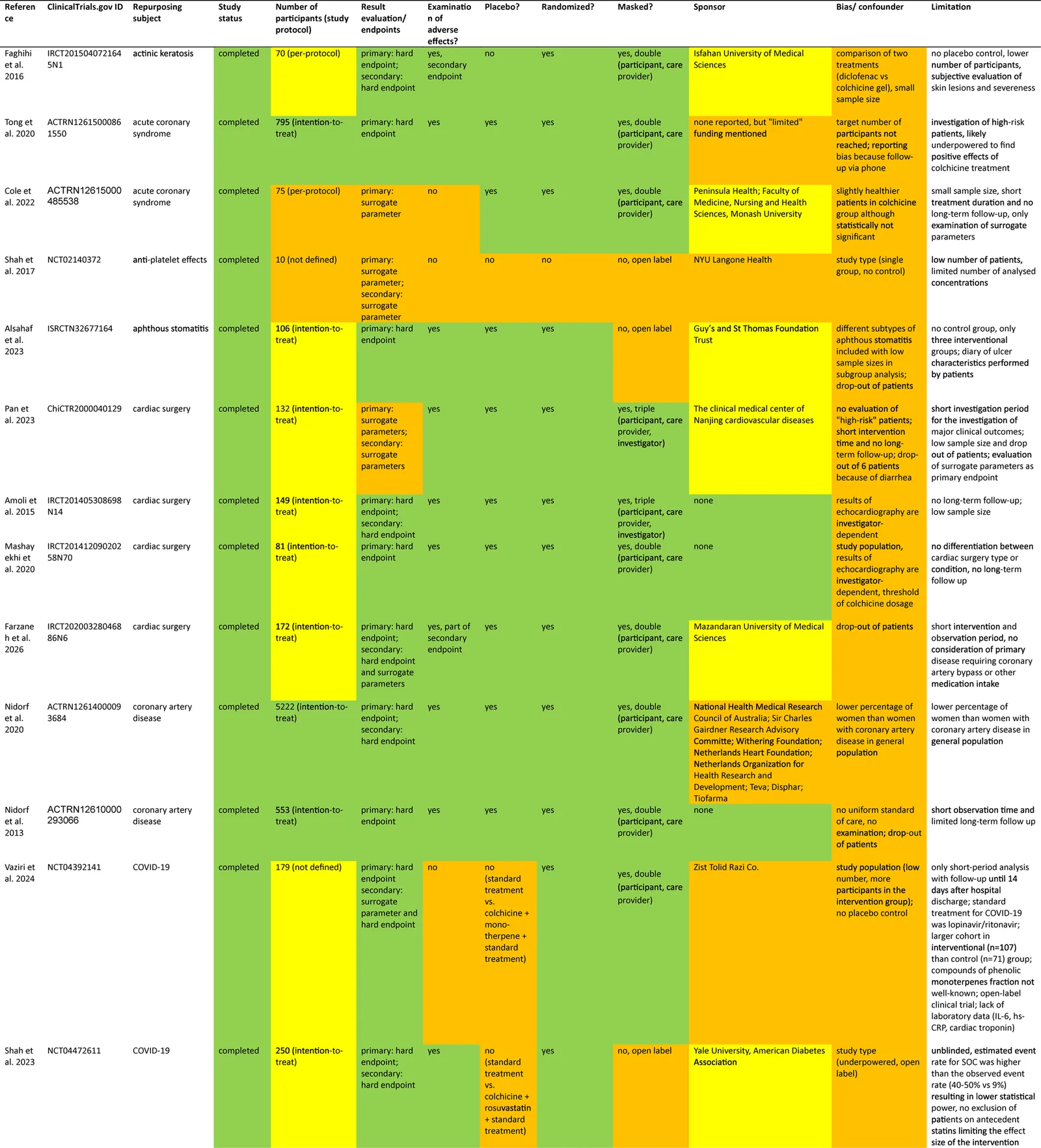

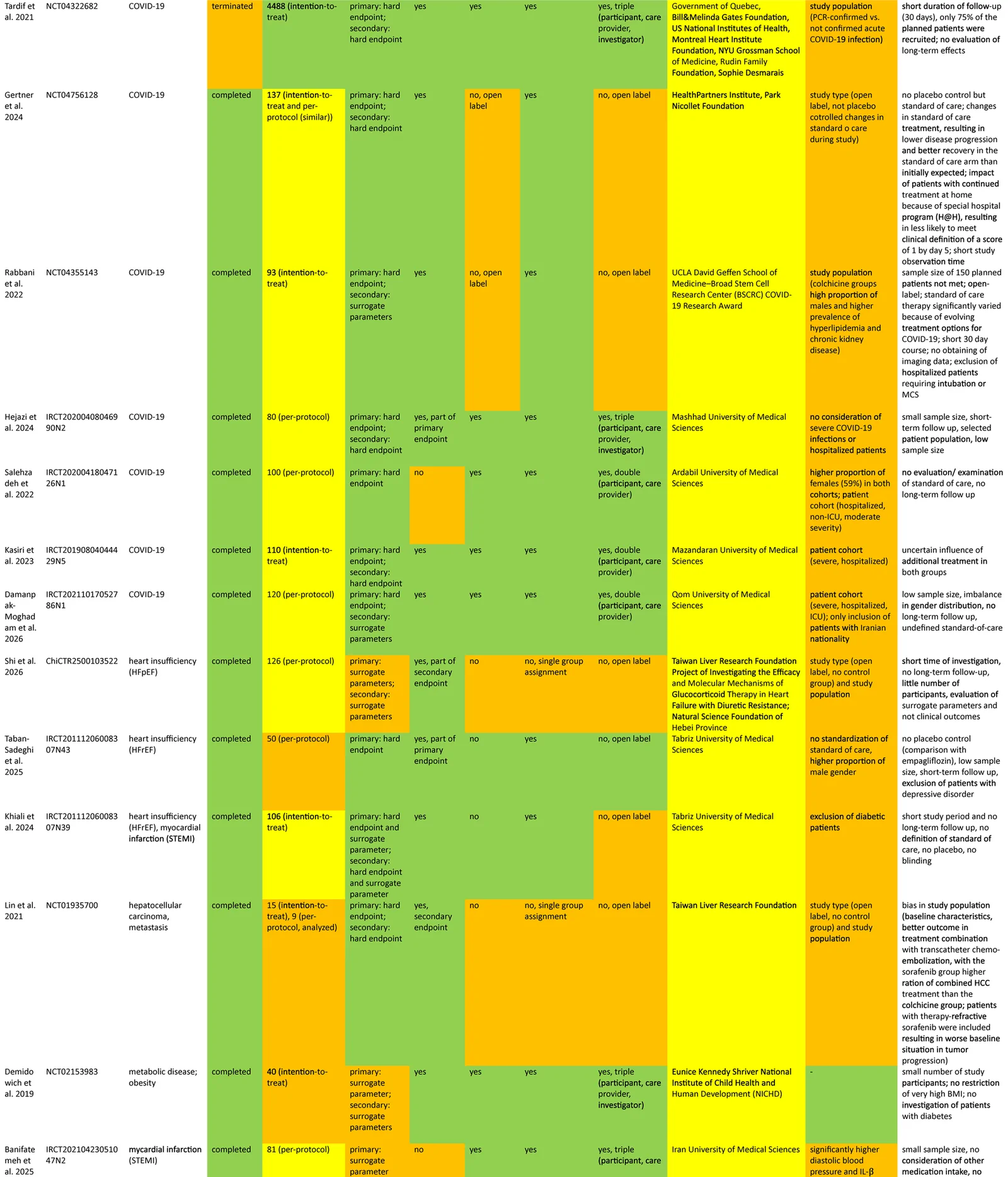

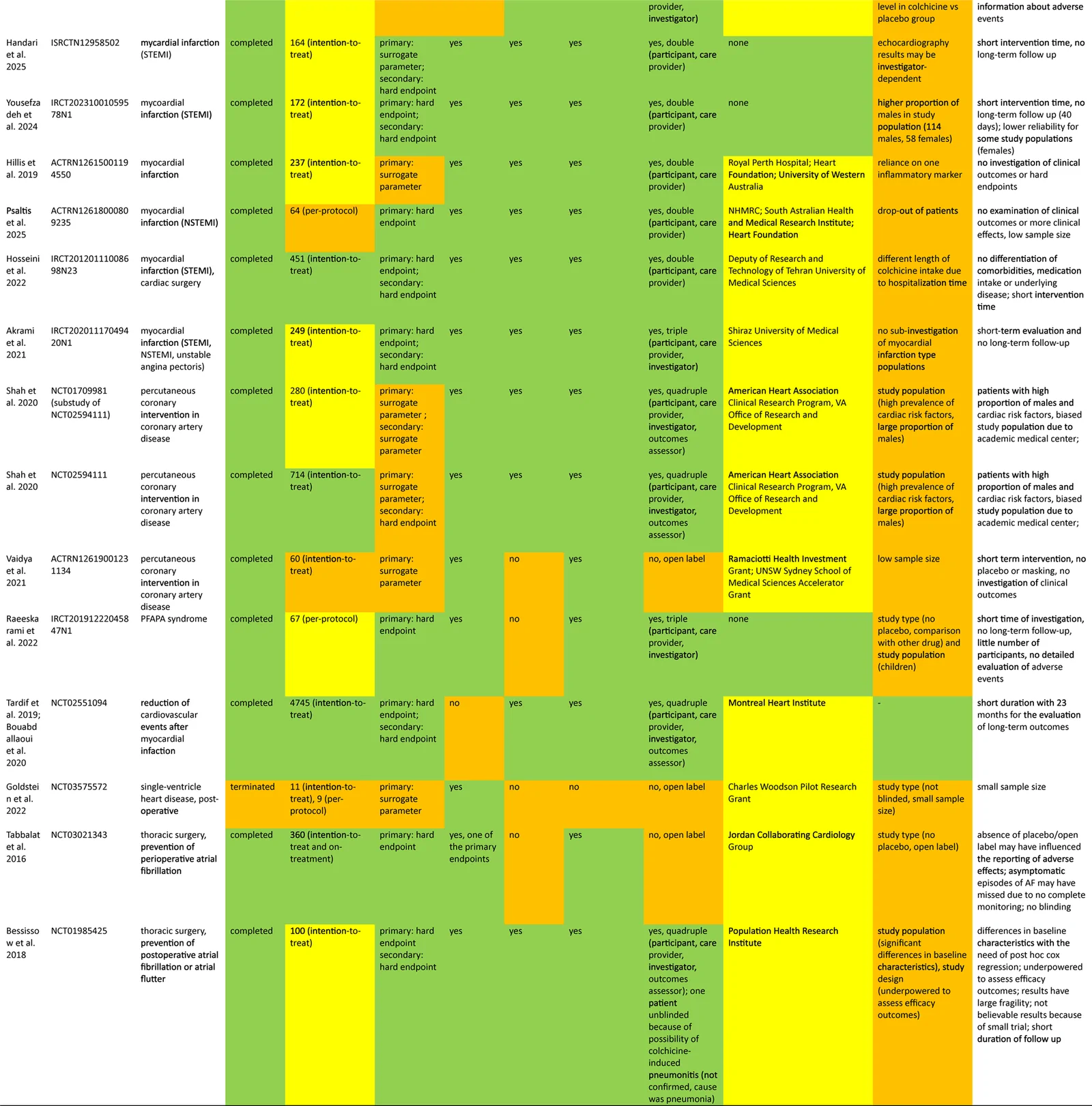
Quality assessment of studies, indicating whether the respective study property increases (green), decreases (orange), or has a neutral effect (yellow) on research quality and value. Additionally, individual study limitations are summarized. An explanation of the assessed aspects can be found in Table S3

In regard to the COVID-19 trials, several methodological characteristics are similar. All studies assessed a hard clinical endpoint, such as mortality, disease severity, or hospitalization duration, which strengthens their clinical relevance. Randomization was applied in all studies, reducing selection bias and improving internal validity. Most studies were completed as planned, with only one early termination [125]. Safety assessments were generally reported, although two trials [108, 136] lacked comprehensive safety documentation [81, 117].

However, notable weaknesses remain [35]. Masking was inconsistent: five studies applied blinding, whereas four were open-label [43, 102, 113, 136], introducing a risk of performance and detection bias. Five of nine studies included a placebo control, increasing the risk of psychological and interpretative bias. Most recruited a moderate sample size (*n* = 80–250), with one larger trial [125], *n* = 4488). Finally, potential bias related to patient selection, severity of COVID-19 infection, hospitalized vs. ambulant treatment, or non-defined and changing standard of care was noted. Taken together, these studies demonstrate methodological strengths in randomization and endpoint selection, but the lack of masking, absence of placebo, and limited sample size in most trials represent key limitations [8, 46, 54, 59, 62].

The 26 trials addressing cardiovascular conditions and interventions showed a more heterogeneous picture in methodological characteristics. All studies, except the trial about single-ventricle heart disease in children [45], were completed, yet their quality varied considerably. Trials in percutaneous coronary intervention (PCI) were generally well-conducted, although they often relied on surrogate endpoints, limiting the clinical applicability of their findings [16, 60]. The heart failure trials were small, open-label or uncontrolled, had short follow-up and mainly evaluated surrogate or functional outcomes, which precludes conclusions about clinical benefit or drug interactions. In thoracic surgery studies, methodological weaknesses such as the absence of masking or comprehensive adverse effect reporting were observed [21, 122, 143]. Particularly concerning were two trials on Fontan patients and antiplatelet effects, which included extremely small patient numbers (around 10), lacked randomization, masking, and placebo control, and thus provided limited evidence. The small sample size can be explained by the characteristics of pilot clinical trials [45, 110], conducted during the COVID-19 pandemic with personnel shortages that hindered study implementation, resulting in the early termination of the Fontan trial, and by safety considerations aimed at obtaining an initial overview of potential effects in healthy participants regarding antiplatelet activity.

The remaining five clinical trials investigating metabolic conditions, HCC and autoinflammatory disorders, displayed mixed methodological rigor. All were completed and four were randomized, but placebo controls were uncommon and sample sizes were small (*n* = 15–106). The HCC study used a single treatment group and a historical control, and the obesity study evaluated a surrogate primary endpoint. Overall, all trial groups share common limitations: small sample sizes in several studies, missing placebo control, and, in some cases, open-label designs, all of which may compromise validity. Nevertheless, strengths such as the frequent use of clinically meaningful endpoints, completion status, and application of randomization in most studies are encouraging. These considerations must be taken into account when interpreting clinical trial results [63, 72, 77, 91, 104, 116, 137, 139].

### Interpretation of evidence for successful repurposing for colchicine in analysed clinical trials

**Fig. 3:**
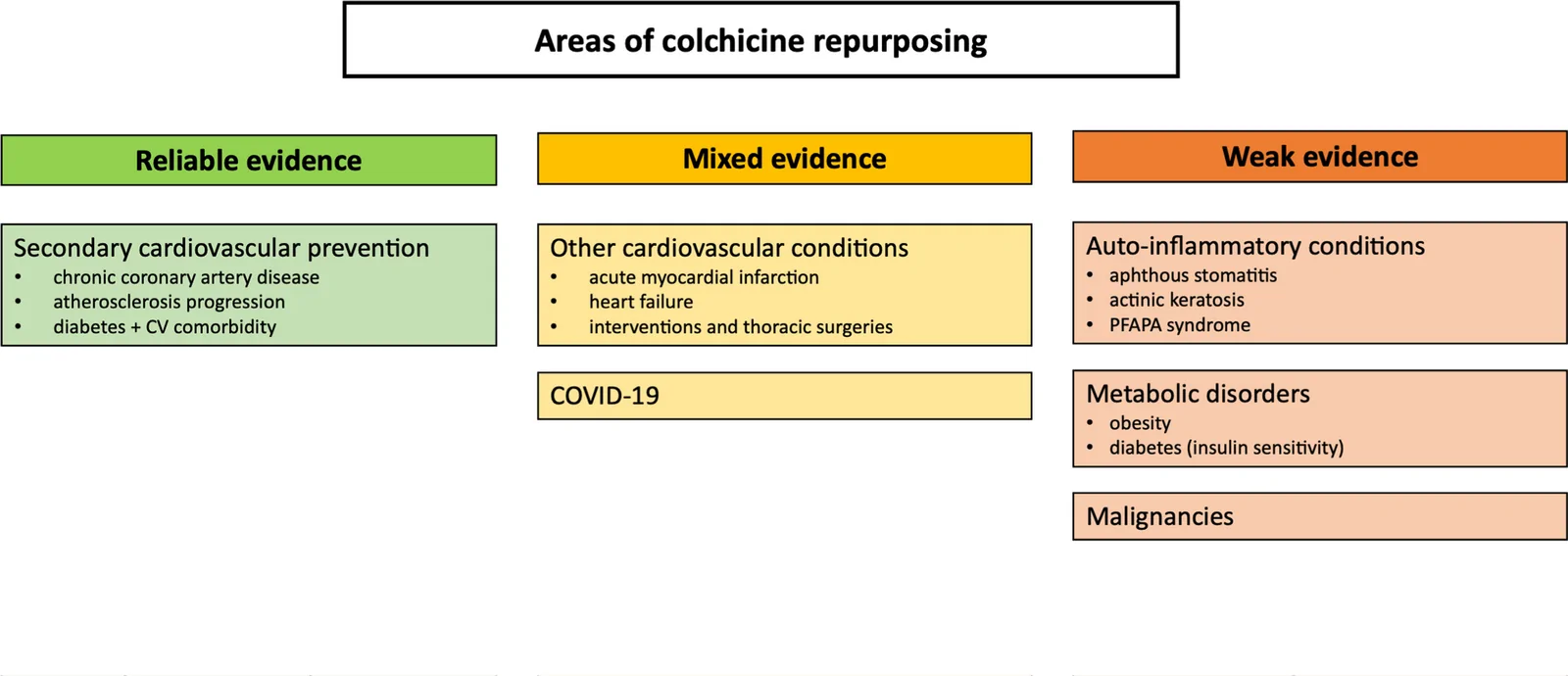
Summary of key findings regarding the repurposing of colchicine. Investigated areas are categorised by their available evidence. Reliable evidence (green) supports its use in secondary cardiovascular prevention, particularly in chronic coronary artery disease, atherosclerosis progression, and the reduction of major adverse cardiovascular events in patients with type 2 diabetes and recent myocardial infarction. Mixed evidence (yellow) was found for other cardiovascular conditions and interventions, as well as COVID-19. Weak evidence (orange) is currently available for autoinflammatory conditions, metabolic disorders, and malignancies. Importantly, clinical trials for a similar indication often report contradicting findings. It is mandatory to consider study design, investigated endpoints and patient population in the interpretation of clinical trials and repurposing potential

Besides the presentation of the findings for each clinical trial individually, it is important to summarize the overall evidence for each repurposing subject. This allows a broader interpretation of the potential benefit and reliability of colchicine, supplemented by insights from a systematic literature search (Fig. 3, Table S4). However, such interpretation requires consideration of the investigated patient populations, clinical settings, and limitations of the included studies (Tables 1 and 2). These factors restrict overall, coherent conclusions.

In COVID-19, three smaller clinical trials found a significant benefit of colchicine in the reduction of mortality, length of hospital stay or fever duration. Reversely, five trials did not find a significant primary benefit. These differing results also appear in the assessment of surrogate parameters, with significant and non-significant changes. Literature findings confirm the contradictory assessment of the benefits of colchicine in COVID-19 infections [5, 13, 25, 33, 44, 52, 69, 71, 144, 146]. The positive subgroup and small-study findings are therefore hypothesis-generating rather than evidence of a consistent overall benefit.

Several factors may explain these inconsistencies. Most COVID-19 trials had a relatively small sample size (*n* = 80–250 patients), except for the large COLCORONA trial with 4488 participants, and there was considerable heterogeneity across studies [44] (Tables 1 and 2). The observed benefit may depend on patient characteristics (age, comorbidities, cardiovascular risk), clinical setting (hospitalized versus outpatient), and disease severity (mild, moderate, severe) [43, 111, 125]. Moreover, many studies assessed colchicine as an add-on therapy to standard of care versus standard of care alone, often without a placebo control. The definition of standard of care was inconsistent and changed during the trial periods due to rapid advances in COVID-19 treatment [43, 113], potentially confounding comparisons within and across trials. However, this may have influenced the whole cohort, with the question whether patients at the beginning vs at the end of the trial are comparable to each other. Furthermore, trials were performed at a time where no vaccination was available [25]. This raises the question of whether patients treated early in a trial were comparable to those treated later. Additionally, these trials were conducted before vaccines became available [25], which limits the applicability to the current situation, where vaccination and improved therapeutic strategies have reduced disease burden. Consequently, repeating such trials today might yield different results, and it is questionable whether this repurposing remains a priority given the current lower clinical and public health impact of COVID-19 [140], the widespread availability of vaccines [141], and established treatment protocols [68]. While unanswered questions remain and further trials could provide clarity [69, 135, 144], the potential benefit of colchicine for COVID-19 appears to be of limited current relevance compared to its importance during the height of the pandemic, when any effective therapy was urgently needed.

For cardiovascular indications, evidence is condition-dependent rather than uniformly promising. The LoDoCo, LoDoCo2 and COLCOT trials support long-term secondary prevention in chronic coronary artery disease and afer myocardial infarction, particularly where inflammatory processes are relevant [4, 4, 10, 30, 32, 66, 86, 88, 88, 89, 89, 90, 100, 145]. In contrast, evidence in acute coronary syndromes, acute myocardial infarction, PCI and cardiac or thoracic surgery is mixed. Several studies did not find a significant clinical benefit or were underpowered [9, 111, 128]. Regarding its antiplatelet effect, trials indicate no relevant impact [110].

Specifically for coronary artery disease and atherosclerosis, repurposing can be considered effective, aligning with the inclusion in international guidelines as an add-on therapy for secondary prevention of cardiovascular events [4, 14, 94]. While its benefit is established in coronary artery disease and pericarditis [14, 23, 105], further evidence is needed for other cardiovascular conditions [19, 30, 42, 66].

For other indications, such as advanced HCC, metabolic disorders, or cutaneous manifestations, current evidence is insufficient to confirm or exclude a benefit and requires further investigation [31, 83]. Literature also highlights other oncologic and hematologic repurposing opportunities, including glioma and clonal haematopoiesis of indeterminate potential (CHIP) or clonal cytopenia of undetermined significance (CCUS) [38, 87]. While most evidence about these cancers remains preclinical, experimental findings suggest possible benefits [38, 85]. In haematology, improvements in surrogate markers have been reported, but these do not necessarily translate into improved clinical outcomes [87], underlining the need for further validation.

In metabolic disease, some inflammatory markers improved with colchicine treatment, but there was no significant effect on the primary measure of insulin sensitivity [28] (Table 1). The trial duration was short (3 months), and only surrogate endpoints were assessed (Tables 1 and 2). However, a COLCOT subgroup analysis in patients with type 2 diabetes and recent myocardial infarction found fewer cardiovascular events in this group [106]. Importantly, this result concerns secondary cardiovascular prevention in patients with diabetes, not improvement of glycaemic control or treatment of diabetes itself. Based on the hypothesis that inflammatory processes contribute to metabolic disorders, colchicine might have a role, but its effect in primary cardiovascular prevention in type 1 and type 2 diabetes and in diabetes prevention requires large-scale, long-term trials.

### Consideration of safety and toxicity

To assess the benefit of a treatment choice, it is essential not only to consider the positive effects but also to take adverse effects into account, resulting in the evaluation of a risk–benefit ratio. For each of the analysed studies, the adverse events are discussed and compared with the literature, followed by a more general assessment. The considerations focus on minor adverse effects, major events, contraindications and precautions, and the risk–benefit ratio.

Minor adverse effects are transient without lasting complications and do not necessarily lead to a stop of drug intake [79, 118]. A significantly higher incidence is often found in gastrointestinal symptoms like diarrhoea [43, 74, 111, 124, 126]. Regarding nausea and vomiting, some trials did not find a significantly higher rate [45], while others did [43]. Gastrointestinal adverse effects are considered the most frequently occurring adverse effects [10, 19, 32, 57, 119, 127]. Another observed effect was anorexia, which occurred at a higher but non-significant rate in the END-AF trial in patients after thoracic surgery [124].

Major adverse effects are more severe complications, can be life-threatening and may result in significant morbidity or death [79, 118]. A higher rate of pneumonia (0.9% vs. 0.4%) was found in the COLCOT trial about cardiovascular conditions [126], while the COVID-19 trial COLCORONA a lower rate was found [125]. A significant increase in AST and ALT was found in a COVID-19 trial, while myalgia had a higher but non-significant prevalence [113]. Literature findings confirm concern about pneumonia risk [32], as well as signals of increased off all-cause or non-cardiovascular death in individual trials, partly related to sepsis [19, 90], and cytopenia or transaminitis [52, 75, 86]. Further concerns are neurotoxicity [38], multiorgan failure [69], myotoxicity, alopecia [41], rhabdomyolysis, gastrointestinal (GI) perforation, as well as renal and hepatic impairment [52]. The immunosuppressive effect is discussed, while some found a correlation between chronic kidney disease and adverse immunosuppression [19], while other literature did not find evidence for this [33].

Cautious use of colchicine is mandatory in patients with decreased kidney and liver function, ranging from dose adjustments to contraindication [93, 107, 112], because drug elimination is delayed, resulting in a higher toxicity and adverse event risk [48]. Colchicine has a narrow therapeutic range, with an abrupt change from therapeutic to toxic effects (Table S5). Overdosing can result in severe adverse effects, and fatal outcomes [44, 75]. Furthermore, there is a high potential for drug interactions [52], particularly with P-glycoprotein (P-gp) and Cytochrome P450 3A4 (CYP3A4)-inhibitors, which can increase the plasma concentration of colchicine [75]. Therefore, it has to be ensured not to use CYP3A4-inhibitors in combination with colchicine. This is particularly problematic in patients with comorbidities as cardiovascular conditions, with several cardiovascular drugs known as acting as CYP3A4-inhibitors, for example verapamil and amiodarone [131]. In pregnancy, there are associations with lower birth weight and increased risk for preterm deliveries, while it is generally considered safe for pregnant women and unborn child [76].

Analysed studies differ in their conclusions about safety and adverse effects. Some studies found a similar rate of adverse events compared to the control group, including the clinical trial about metabolic disease [28] and the COP-AF pilot trial [9]. In contrast, other trials found a significantly higher rate of adverse events, resulting in withdrawal of participants [102, 124]. Although colchicine toxicity is dose-dependent, comparisons across trials cannot establish a dose–response relationship because dosing regimens, conditions, populations and follow-up differed (Table S6). Literature assesses the safety profile as predominantly favourable at low doses [88, 89, 135], while others highlight that benefits and risks have to be balanced [86]. It is often stressed that colchicine’s properties, adverse effects and toxicity are well-known [32, 33, 87], with minor adverse effects than some other anti-inflammatory drugs [88, 89]. However, the high potential for toxic drug interactions is a challenge in repurposing [19], potentially limiting its use in comorbid patients [14, 78].

## Limitations

This study has several limitations. only registered trials with a peer-reviewed publication and extractable results were included. Ongoing, unpublished and registry-only studies were excluded, which introduces availability and publication bias. Search and filtering functions differed between registries, manual screening was required for ChiCTR and IRCT, and cross-registration complicated deduplication. The registry search was conducted on July 07 2026 and the literature search on July 08 2026, data published later were not considered. The literature search was limited to PubMed and Scopus, and the specific term “colchicine AND repurposing” may have missed suitable publication that did not use repurposing terminology. The review was not preregistered, which increases the risk of deviations from methods decided in advance. Finally, the assessment of study quality characteristics by predefined aspects provides a useful overview, but was not a formal risk-of bias assessment. For final conclusions, individual study settings and purposes must be considered.

## Conclusions and further perspectives

Colchicine, a drug used for many years primarily for gout, has experienced new popularity due to its recognized potential in a variety of further conditions [26]. These repurposing subjects span multiple organ systems and conditions [75, 120]. Their common ground is the involvement of inflammatory processes [41, 90, 144], which are suppressed and reduced by colchicine [88, 89]. This provides a biological rationale for investigating colchicine in vascular [74, 93, 130], metabolic [15, 29], infectious [34], neoplastic [65, 73, 74], and autoimmune [18, 70, 109] conditions. However, biological plausibility alone does not imply clinical benefit.

Swift, comprehensive and transparent publication of data is essential for informing the research community and advancing development [55, 115]. The lack of results for often more than half of the registered trials has also been reported in other publications, raising concerns about good scientific practice and wasting of research [37, 55, 115]. Only studies of high quality and with representative populations can be generalized and add value to the study landscape [56, 134]. However, every clinical trial has to be critically examined so that the results can be contextualised and their implications interpreted correctly, since every clinical trial has its own profile of characteristics and limitations, making its findings restricted or relevant to a particular patient group, condition, and setting [43, 80, 97, 111, 125]. It is therefore important to design and conduct high-quality studies [138], with a sufficient number of randomized participants, a blinded and placebo-controlled comparison, the consideration of hard endpoints, and the monitoring of adverse events (Table S3).

The principle of repurposing established drugs is valuable, because these approved drugs are already developed and clinically observed [27, 64], including knowledge about safety, adverse effects and contraindications [87]. This can decrease the cost and time required for drug development and make treatment improvements available to patients more rapidly [99, 133]. For colchicine, however, the evidence is indication-specific. Low-dose colchicine is supported for secondary cardiovascular prevention in coronary artery disease, and has been approved for cardiovascular risk reduction in adult patients with atherosclerotic disease or multiple risk factors [4, 93]. The current evidence does not support a broad benefit across cardiovascular disease. Evidence for COVID-19, metabolic, oncologic, cutaneus and autoinflammatory indication remains insufficient or contradictory. Further high-quality clinical trials are needed to validate benefits while carefully considering adverse effects and drug interactions [67].

Further research is required to update clinical trial findings on colchicine when new data become available, both regarding new indications and its safety profile. Priority should be given to well-powered trials with clinical endpoints in areas where the current evidence remains uncertain, particularly for heart failure and other acute cardiovascular settings.

## Supplementary information

Below is the link to the electronic supplementary material.

Supplementary file1 (DOCX 92 KB)

## Data Availability

All source data for this study are available upon reasonable request from the authors.
