# Supplementary material for: Repurposing of colchicine: critical examination of clinical trials and scoping review
Source: Eur J Clin Pharmacol. 2026 Aug 20;82(9):238. doi: 10.1007/s00228-026-04149-w (PMC13490232; doi:10.1007/s00228-026-04149-w)
Supplement: Supplementary file 1 [file 228_2026_4149_MOESM1_ESM.docx]

**Repurposing of colchicine:**

**critical examination of clinical trials and scoping review**

**Supplement**

**Lilly Josephine Bindel and Roland Seifert**

Supplemental Results

**Repurposing for COVID-19 infections**

Nine studies investigated the benefit of colchicine in acute COVID-19 infections, designed as an add-on therapy to standard of care, for short-term effects. Eight studies were completed, while one was terminated (COLCORONA trial). Some studies tested colchicine in combination with other substances, while others evaluated colchicine alone. An overview of results can be found in Table 1.

The COLCORONA trial (Tardif et al. 2021), NCT04322682, was an interventional, prospective, randomized, double-blind, placebo-controlled, multi-centre study started in 2020. It focused on patients with COVID-19 infection and high-risk characteristics in an ambulant setting. A total of 4488 patients were included. The primary endpoint was the rate of death and lung complications related to COVID-19, while the secondary endpoint assessed treatment safety compared to placebo. The primary endpoint (reduction of mortality and lung complications for colchicine vs. placebo) was not significant in the total study population (OR 0.79, CI 0.61–1.03, p=0.081) but was statistically significant in PCR-confirmed patients (OR 0.75, CI 0.57–0.99, p=0.042). Adverse effects were not significantly increased in the colchicine group compared to placebo (4.9% vs. 6.3%, p=0.051).

The COLTREXONE trial (Gertner et al. 2024), NCT04756128, was an interventional, prospective, randomized, open-label, multi-centre study started in 2021. It included 137 hospitalized participants with moderate COVID-19 and investigated four arms, consisting of standard of care alone, colchicine plus standard of care, naltrexone plus standard of care, and the combination of colchicine and naltrexone plus standard of care. The primary endpoint was COVID-19 severity, based on the time required to reach a clinical scale score of 1 from enrolment to the end of hospitalization. Secondary endpoints included total hospitalization duration, number of remdesivir and glucocorticoid receptor (GCR) agonist doses in required patients, and the need for high-flow nasal cannula (HFNC), non-invasive positive pressure ventilation (NIPPV), or intensive care unit (ICU) care. Outcomes showed no treatment effect, with no significant differences in any primary or secondary endpoints (p>0.05).

The trial of Rabbani et al. (2022), NCT04355143, was an interventional, prospective, randomized, open-label, multi-centre study started in 2020. It included 93 hospitalized patients with COVID-19 infection and evidence of cardiac injury, comparing colchicine plus standard of care with standard of care alone. Primary endpoints included mortality rates, need for mechanical ventilation, and circulatory support, while secondary endpoints assessed changes in inflammatory biomarkers. Neither primary nor secondary outcomes showed significant differences, with no difference in reaching the composite primary endpoint (19% colchicine vs. 15% standard of care, p=0.78).

The trial of Shah et al. (2023), NCT04472611, was an interventional, prospective, randomized, open-label, multi-centre study started in 2020. It compared colchicine combined with rosuvastatin plus standard of care against standard of care alone. The trial included 250 hospitalized participants with COVID-19 infection who were not critically ill. Primary endpoints included COVID-19 severity (defined by the WHO ordinal scale, ranging from 1 to 8) and thromboembolic events. Secondary endpoints were mortality rate, respiratory failure requiring intubation, and myocardial injury. No statistically significant differences were observed in any primary or secondary endpoints (p>0.05), and no reduction in COVID-19 severity.

The trial of Vaziri et al. (2024), NCT04392141, was an interventional, prospective, randomized, double-blind, single-centre study started in 2020. It compared colchicine combined with phenolic monoterpenes and standard of care against standard of care alone. A total of 179 hospitalized participants with COVID-19 infection were included. The primary endpoint was the mortality rate, and secondary endpoints included SpO₂ levels, lymphocyte count, serum lactate dehydrogenase (LDH), and length of hospitalization. The primary endpoint was achieved, showing a significant reduction in mortality (0.9% vs. 8.4%, p=0.035), whereas secondary endpoints were not significantly improved.

The trial of Hejazi et al. (2024), IRCT20200408046990N2, was an interventional, prospective, randomized, triple-blind, placebo-controlled, single-centre study started in 2020. It included 80 non-hospitalized participants with mild to moderate COVID-19 infection, comparing colchicine with placebo. The primary endpoints were treatment response and adverse drug reactions, while secondary endpoints included duration of clinical symptoms and other clinical outcomes. No significant differences were observed in the duration of symptoms, CRP, lymphopenia, fever, or cough (p>0.05). Colchicine was well tolerated, and no serious adverse effects were reported.

The trial of Salehzadeh et al. (2022), IRCT20200418047126N1, was an interventional, prospective, randomized, double-blind, placebo-controlled, single-centre study started in 2020. It included 100 hospitalized participants with COVID-19 infection, comparing colchicine plus standard of care with placebo plus standard of care. The primary endpoints were duration of hospitalization and fever cessation. Fever persisted for a significantly shorter time, and hospitalization duration was significantly reduced in the colchicine group. Improvement of dyspnoea did not differ significantly between groups, and adverse effects were not reported.

The trial of Kasiri et al. (2023), IRCT20190804044429N5, was an interventional, prospective, randomized, double-blind, placebo-controlled, single-centre study started in 2021. It included 110 hospitalized participants with COVID-19 infection. Both groups received interferon beta-1b and remdesivir, while colchicine or placebo was additionally administered. The primary endpoint was clinical response, and secondary endpoints included hospital complications and 28-day mortality. No significant differences were observed in treatment response, mortality, ICU admission, treatment complications, or hospitalization duration (p>0.05). Diarrhoea and nausea were the major adverse effects.

The trial of Damanpak-Moghadam et al. (2026), IRCT20211017052786N1, was an interventional, prospective, randomized, double-blind, placebo-controlled, single-centre study started in 2022. It included 120 hospitalized participants with severe COVID-19 infection who were intubated and connected to a ventilator. Primary endpoints included mortality and length of hospital stay, while secondary endpoints assessed inflammatory markers. Mortality and hospitalization duration were significantly lower in the colchicine group. LDH levels differed significantly, whereas no significant differences were observed in WBC, CRP, or lymphocyte counts. No severe adverse events were reported.

**Repurposing for cardiovascular conditions and thoracic interventions**

Twenty-five clinical studies investigated the benefit of colchicine in cardiovascular conditions and interventional settings, while one study assessed its effects on inflammatory markers. One clinical study assessed its anti-platelet effect in healthy adults. Some trials were open-label without placebo or control groups, while others included placebo comparisons. Most studies were completed, and one was terminated. An overview of results can be found in Table 1.

The COLCHICINE-PCI trial and its substudy (NCT02594111 and NCT01709981) were interventional, prospective, randomized, double-blind, placebo-controlled, multi-centre studies initiated in 2013. They evaluated colchicine in PCI patients with ischemic heart disease or acute coronary syndrome (n=714 and n=280). The main study’s primary outcome was troponin I levels 24 hours post-PCI, and the secondary outcome was the occurrence of major adverse cardiovascular events (MACE). The substudy focused on interleukin (IL)-6 and IL-1β changes one hour after PCI as the primary endpoint, with IL-6 change at 24 hours as a secondary outcome. Neither study showed a significant reduction in primary outcomes. In the sub study, one secondary endpoint (IL-6 at 24 hours) was significant, whereas all others were not.

The COLCOT trial (Tardif et al. 2019; Bouabdallaoui et al. 2020), NCT02551094, was an interventional, prospective, randomized, double-blind, placebo-controlled, multi-centre study initiated in 2015. It investigated whether colchicine could reduce secondary cardiovascular events after MI in 4745 participants. Primary and secondary endpoints included cardiovascular death, resuscitated cardiac arrest, MI, stroke, or urgent hospitalization for angina. A significant reduction in the risk of ischemic and cardiovascular events was observed (5.5% colchicine vs. 7.1% control, HR 0.77, CI 0.61–0.96, p=0.02).

The COP-AF pilot trial (Bessissow et al. 2018), NCT01985425, was a randomized, double-blind, placebo-controlled, multi-centre study initiated in 2014, including 100 participants undergoing lung resection surgery. It investigated whether colchicine prevented postoperative atrial fibrillation or atrial flutter. The primary endpoint was new atrial fibrillation lasting longer than 30 seconds or resulting in a cardiac event. Secondary endpoints included death, new-onset atrial flutter, myocardial injury after non-cardiac surgery (MINS), stroke, transient ischemic attack (TIA), and postoperative infection. Adjusted outcomes did not confirm a significant reduction in risk for any primary or secondary endpoint (p>0.05).

The END-AF trial (Tabbalat et al. 2016), NCT03021343, was an interventional, prospective, randomized, open-label, multi-centre study initiated in 2012. In 360 participants undergoing open-heart surgery, it investigated whether colchicine prevented perioperative atrial fibrillation compared to control. Endpoints included the rate of atrial fibrillation until discharge and adverse events. AF occurred less frequently in the colchicine group (14.5% vs. 20.5%), but the difference was not significant (p=0.14). Adverse effects were more common in the colchicine group, diarrhoea was significantly higher (24.6% vs. 5.5%, p<0.001), while anorexia was not (n=11 vs. n=4, p=0.07).

The trial of Goldstein et al. (2022), NCT03575572, was an interventional, prospective, single-group assignment with historical control, single-centre study initiated in 2018. It included 11 children to investigate whether colchicine benefited postoperative Fontan patients with single-ventricle heart disease. The primary endpoint was pro-inflammatory cytokine levels, and secondary endpoints included length of hospitalization, chest tube duration, and cytokine levels. No significant reduction in cytokine levels was observed between colchicine and control groups (p>0.05). For secondary endpoints, significant reductions in hospital stay and chest tube duration were found in the per-protocol population but not in the intention-to-treat population.

The trial of Shah et al. (2017), NCT02140372, was an interventional, prospective, single-centre trial initiated in 2014. It examined the anti-platelet effects of colchicine in healthy adults without a control group. The primary endpoint was monocyte–platelet aggregation between baseline and 2 hours after treatment. Secondary endpoints included light transmission aggregometry for adenosine diphosphate and epinephrine and platelet adhesion at baseline vs. 2 hours later. Results showed no significant reduction in platelet aggregation after 2 hours (p=0.45) or 24 hours (p=0.65).

The COPS trial (Tong et al. 2020), ACTRN12615000861550, was an interventional, prospective, randomized, double-blind, placebo-controlled study initiated in 2015. It included 795 participants with acute coronary syndrome undergoing PCI or medical therapy. The primary endpoint was a composite of all-cause mortality, acute coronary syndrome, ischemia-driven urgent revascularization, and non-cardioembolic ischemic stroke. Colchicine did not significantly improve the primary cardiovascular outcome. Total and non-cardiovascular death occurred more frequently in the colchicine group, while reported adverse effect rates did not differ between colchicine and placebo.

The COPE-PCI trial (Cole et al. 2022), ACTRN12615000485538, was an interventional, prospective, randomized, double-blind, placebo-controlled, single-centre study initiated in 2017. It included 75 participants with acute coronary syndrome undergoing PCI or medical therapy. The primary endpoint assessed inflammatory biomarkers. Measures of inflammation were similar between treatment groups, while the absolute change in troponin was lower in participants treated with colchicine. The study therefore showed significant and non-significant effects among the investigated surrogate parameters. Adverse effects were not reported.

The trial of Pan et al. (2023), ChiCTR2000040129, was an interventional, prospective, randomized, triple-blind, placebo-controlled, single-centre study initiated in 2020. It included 132 participants undergoing cardiovascular surgery with cardiopulmonary bypass. The primary endpoint was cardiac troponin T, while secondary endpoints included other myocardial injury and inflammatory biomarkers, 30-day mortality, mechanical ventilation, stroke, and complications. Cardiac troponin T, cardiac troponin I, and CK-MB were significantly reduced, as was post-pericardiotomy syndrome. Other major adverse events did not differ. Diarrhoea occurred in 9.2% of participants receiving colchicine.

The trial of Amoli et al. (2015), IRCT201405308698N14, was an interventional, prospective, randomized, triple-blind, placebo-controlled, single-centre study initiated in 2014. It included 149 participants undergoing open cardiac surgery. The primary endpoint was the decrease in pericardial effusion volume assessed by echocardiography. No significant differences were observed between colchicine and placebo regarding pre- and post-treatment pericardial effusion size, changes in effusion, or surgical complications (p>0.05). No significant drug-related adverse effects were reported.

The trial of Mashayekhi et al. (2020), IRCT20141209020258N70, was an interventional, prospective, randomized, double-blind, placebo-controlled, single-centre study initiated in 2013. It included 81 participants undergoing open-heart surgery. Primary outcomes included the occurrence of atrial fibrillation and pericardial effusion. Post-pericardiotomy syndrome occurred significantly less often in the colchicine group. Differences were not significant immediately or one week after surgery but became significant after one month and during follow-up at three and six months. No serious drug-related adverse effects were reported.

The trial of Farzaneh et al. (2026), IRCT20200328046886N6, was an interventional, prospective, randomized, double-blind, placebo-controlled, single-centre study initiated in 2024. It included 172 participants undergoing coronary artery bypass graft surgery. The primary endpoint was the incidence of postoperative atrial fibrillation (POAF), while secondary endpoints included early and late POAF, other arrhythmias, inflammatory markers, hospitalization duration, and adverse events. Colchicine significantly reduced POAF, with an absolute risk reduction of 29.0%, including early and late POAF. Other arrhythmias were not significantly reduced. Gastrointestinal adverse events, particularly diarrhoea, were common but manageable, and no serious adverse events occurred.

The LoDoCo2 trial (Nidorf et al. 2020), ACTRN12614000093684, was an interventional, prospective, randomized, double-blind, placebo-controlled study initiated in 2016. It included 5222 participants with stable coronary artery disease. The primary endpoint was a composite of cardiovascular death, spontaneous MI, ischemic stroke, and ischemia-driven coronary revascularization. The secondary endpoint excluded coronary revascularization. The primary endpoint was significantly reduced with colchicine, whereas the secondary endpoint was not. Death from non-cardiovascular causes occurred more frequently in the colchicine group, but the difference was not significant. No serious adverse events were associated with colchicine.

The LoDoCo trial (Nidorf et al. 2013), ACTRN12610000293066, was an interventional, prospective, randomized study initiated in 2008. It included 553 participants with stable coronary artery disease. The primary endpoint was a composite of acute coronary syndrome, out-of-hospital cardiac arrest, and non-cardioembolic ischemic stroke. The composite primary event occurred significantly less frequently in participants treated with colchicine than in the control group. Thirty-two participants (11%) discontinued colchicine because of intestinal intolerance.

The trial of Shi et al. (2026), ChiCTR2500103522, was a prospective, observational, open-label, single-group study initiated in 2024. It included 126 participants with heart failure with preserved ejection fraction (HFpEF) and elevated inflammatory factors. Primary endpoints included inflammatory markers, while secondary endpoints assessed renal and liver function, depression, anxiety, quality of life, and blood parameters. IL-6 and IL-8 levels decreased significantly, whereas IL-2 and TNF-α did not. Quality of life, depression, and anxiety scores improved significantly. Adverse reactions occurred in 4.1%, were mainly mild gastrointestinal effects, and included transient decreases in eGFR.

The trial of Taban-Sadeghi et al. (2025), IRCT20111206008307N43, was an interventional, prospective, randomized, open-label, active-comparison study initiated in 2022. It included 50 participants with heart failure with reduced ejection fraction (HFrEF), comparing colchicine with empagliflozin in addition to standard of care. The primary endpoint was the Hamilton Depression Rating Scale at baseline, week 4, and week 12, together with adverse event occurrence. Depression scores decreased significantly from baseline in both groups, but no significant difference was observed between groups. Treatment was well tolerated, and no serious adverse effects were reported.

The trial of Khiali et al. (2024), IRCT20111206008307N39, was an interventional, prospective, randomized, open-label study initiated in 2022. It included 106 participants with HFrEF after recent STEMI. The three groups received high-dose empagliflozin, low-dose empagliflozin combined with colchicine, or low-dose empagliflozin, in addition to standard of care. Primary endpoints were NYHA functional class and hsCRP, while secondary endpoints included inflammatory markers and LVEDD. No significant differences were observed between groups. hsCRP decreased significantly in all groups, and neither colchicine nor high-dose empagliflozin was superior to low-dose empagliflozin. No major adverse effects occurred.

The trial of Banifatemeh et al. (2025), IRCT20210423051047N2, was an interventional, prospective, randomized, triple-blind, placebo-controlled, single-centre study initiated in 2022. It included 81 participants with STEMI. The primary endpoint assessed inflammatory markers, while the secondary endpoint was the occurrence of recurrent MI. Inflammatory markers decreased significantly in the colchicine group compared with both baseline and placebo. Information about adverse effects was not provided.

The trial of Handari et al. (2025), ISRCTN12958502, was an interventional, prospective, randomized, double-blind, placebo-controlled study initiated in 2022. It included 164 participants with STEMI. Primary endpoints were Galectin-3 and PIIINP levels, while secondary endpoints included changes in left ventricular end-diastolic volume (LVEDV) and MACE. Galectin-3 and PIIINP levels were significantly reduced after five days, but PIIINP was not significantly reduced within the first 24 hours. No significant differences were observed in LVEDV changes or MACE incidence. No severe adverse events occurred.

The trial of Yousefzadeh et al. (2024), IRCT20231001059578N1, was an interventional, prospective, randomized, double-blind, placebo-controlled, single-centre study initiated in 2022. It included 172 participants with STEMI. Primary endpoints included severity of clinical symptoms, functional capacity, and rehospitalization, while secondary endpoints included echocardiographic measures, LVEF, and left ventricular thrombosis. No significant differences were observed in readmission, functional capacity, LVEDD, LVEF, CRP, atrial fibrillation, or left ventricular thrombosis. Diarrhoea occurred more frequently with colchicine, but overall adverse effects did not differ significantly, and no serious adverse events occurred.

The LoDoCo-MI trial (Hillis et al. 2019), ACTRN12615001194550, was an interventional, prospective, randomized, double-blind, placebo-controlled study initiated in 2016. It included 237 participants with acute type 1 MI. The primary endpoint was the proportion of participants with residual CRP levels exceeding 2 mg/L after 30 days of treatment. Colchicine did not significantly reduce absolute CRP levels or the proportion of participants above the CRP threshold compared with placebo. Treatment was well tolerated, and no serious adverse effects were reported.

The COCOMO-ACS trial (Psaltis et al. 2025), ACTRN12618000809235, was an interventional, prospective, randomized, double-blind, placebo-controlled study initiated in 2018. It included 64 participants with acute non-ST-elevation myocardial infarction (NSTEMI). The primary endpoint was the relative and absolute change in minimum fibrous cap thickness. Colchicine did not significantly affect changes in minimum fibrous cap thickness compared with placebo. Adverse events did not occur more frequently in the colchicine group.

The PodCAST-PCI trial (Hosseini et al. 2022), IRCT20120111008698N23, was an interventional, prospective, randomized, double-blind, placebo-controlled, single-centre study initiated in 2018. It included 451 participants with acute STEMI undergoing PCI. The primary endpoint was the no-reflow phenomenon, while secondary endpoints included ST resolution 90 minutes after PCI, myocardial injury and inflammatory markers, predischarge ejection fraction, and MACE after one month and one year. No significant differences were observed in no-reflow rates, biomarkers, MACE, or predischarge ejection fraction. No major adverse effects occurred.

The trial of Akrami et al. (2021), IRCT20201117049420N1, was an interventional, prospective, randomized, triple-blind, placebo-controlled, single-centre study initiated in 2019. It included 249 participants after STEMI, NSTEMI, or unstable angina pectoris. Primary and secondary endpoints included mortality, ischemic stroke, hospitalization for chest pain, urgent revascularization, decompensated heart failure, cardiac arrest, MI, and MACE. MACE occurred significantly less frequently, and survival was significantly higher with colchicine, while laboratory markers did not differ significantly. Gastrointestinal adverse effects were significantly more frequent, and two participants discontinued treatment because of diarrhoea.

The trial of Vaidya et al. (2021), ACTRN12619001231134, was an interventional, prospective, randomized, open-label study initiated in 2015. It included 60 participants with acute coronary syndrome undergoing PCI and mechanistically assessed inflammatory markers. Significant reductions were observed in neutrophil extracellular trap (NET) and neutrophil elastase (NE) release under some assessed conditions, whereas myeloperoxidase and other NET or NE measurements were not significantly changed. No adverse events were reported.

**Repurposing for further conditions**

The trial by Demidowich et al. (2019), NCT02153983, was an interventional, prospective, randomized, double-blind, placebo-controlled, single-centre study initiated in 2014. It investigated 40 obese patients without diabetes, comparing colchicine with control for effects on metabolic processes, including diabetes risk. The primary endpoint was change in insulin sensitivity by frequently sampled intravenous glucose tolerance test (FSIVGTT), while secondary endpoints included changes in fasting insulin, homeostatic model assessment on insulin resistance (HOMA-IR index), C-reactive protein (CRP), and other inflammatory markers from baseline to three months. The primary endpoint showed no significant difference (FSIVGTT mean 0.29 colchicine vs. 0.34 control, p=0.82). Among secondary endpoints, significant reductions were observed in hsCRP (p=0.0015), ESR (p=0.007), and HOMA-IR (p=0.0499), whereas fasting insulin (p=0.07), acute insulin response to glucose (AIRg), haemoglobin A1c, and HDL-/LDL-cholesterol were not significantly reduced (p>0.05).

The trial by Lin et al. (2021), NCT01935700, was an interventional, prospective, open-label, single-group assignment, single-centre study initiated in 2013. It investigated nine patients with advanced HCC (TNM stage 3b–4b) for potential benefit of colchicine, compared to a historical control group treated with sorafenib. The primary endpoint was overall survival, and the secondary endpoint was the occurrence of grade 3 severe adverse events, assessed up to 72 months. Outcomes did not show a survival benefit (p>0.2). Mild adverse effects were common, with biliary tract obstruction (p=0.018) and diarrhoea (p<0.05) being significantly more frequent in the colchicine group (Table 1).

The trial of Faghihi et al. (2016), IRCT2015040721645N1, was an interventional, prospective, randomized, double-blind, active-comparison, single-centre study initiated in 2013. It included 70 participants with actinic keratosis and investigated 1% colchicine gel. The primary endpoint assessed skin lesions and recovery, while the secondary endpoint assessed adverse effects. Both treatment groups improved, but no significant differences were observed in lesion size, recovery, or adverse effect occurrence.

The trial of Alsahaf et al. (2023), ISRCTN32677164, was an interventional, prospective, randomized, open-label, three-arm study initiated in 2010. It included 106 participants with recurrent aphthous stomatitis and compared betamethasone mouthwash, colchicine tablets, and their combination. The primary endpoint was the ulcer severity score. Mean ulcer severity decreased significantly in all three treatment groups, but no significant differences were observed between groups. Adverse effects were significantly more frequent with colchicine and resulted in participant withdrawal. Reported effects included gastric pain, peripheral neuropathy, and alopecia.

The trial of Raeeskarami et al. (2022), IRCT20191222045847N1, was an interventional, prospective, randomized, triple-blind, active-comparison, single-centre study initiated in 2019. It included 67 children aged one to 10 years with PFAPA syndrome. Both groups received prednisolone and were additionally treated with colchicine or cimetidine. The primary endpoint was the number of febrile episodes and days without fever. Febrile episodes decreased significantly in both groups, but no significant difference was observed between colchicine and cimetidine. No serious adverse events were reported.

Supplemental Tables

***Table S1:*** *Preferred Reporting Items for Systematic reviews and Meta-Analyses extension for Scoping Reviews (PRISMA-ScR) Checklist (Tricco et al. 2018).*

| **SECTION** | **ITEM** | **PRISMA-ScR CHECKLIST ITEM** | **REPORTED ON PAGE #** |
| --- | --- | --- | --- |
| **TITLE** | | | |
| Title | 1 | Identify the report as a scoping review. | 1 |
| **ABSTRACT** | | | |
| Structured summary | 2 | Provide a structured summary that includes (as applicable): background, objectives, eligibility criteria, sources of evidence, charting methods, results, and conclusions that relate to the review questions and objectives. | 2 |
| **INTRODUCTION** | | | |
| Rationale | 3 | Describe the rationale for the review in the context of what is already known. Explain why the review questions/objectives lend themselves to a scoping review approach. | 2-3 |
| Objectives | 4 | Provide an explicit statement of the questions and objectives being addressed with reference to their key elements (e.g., population or participants, concepts, and context) or other relevant key elements used to conceptualize the review questions and/or objectives. | 3 |
| **METHODS** | | | |
| Protocol and registration | 5 | Indicate whether a review protocol exists; state if and where it can be accessed (e.g., a Web address); and if available, provide registration information, including the registration number. | No protocol registered |
| Eligibility criteria | 6 | Specify characteristics of the sources of evidence used as eligibility criteria (e.g., years considered, language, and publication status), and provide a rationale. | 3-4 |
| Information sources* | 7 | Describe all information sources in the search (e.g., databases with dates of coverage and contact with authors to identify additional sources), as well as the date the most recent search was executed. | 3-4 |
| Search | 8 | Present the full electronic search strategy for at least 1 database, including any limits used, such that it could be repeated. | 3-4 |
| Selection of sources of evidence† | 9 | State the process for selecting sources of evidence (i.e., screening and eligibility) included in the scoping review. | 3-4 |
| Data charting process‡ | 10 | Describe the methods of charting data from the included sources of evidence (e.g., calibrated forms or forms that have been tested by the team before their use, and whether data charting was done independently or in duplicate) and any processes for obtaining and confirming data from investigators. | 3-4 |
| Data items | 11 | List and define all variables for which data were sought and any assumptions and simplifications made. | 3-4; 10-11 |
| Critical appraisal of individual sources of evidence | 12 | If done, provide a rationale for conducting a critical appraisal of included sources of evidence; describe the methods used and how this information was used in any data synthesis (if appropriate). | 3-4; 10-11 |
| Synthesis of results | 13 | Describe the methods of handling and summarizing the data that were charted. | 3-4 |
| **RESULTS** | | | |
| Selection of sources of evidence | 14 | Give numbers of sources of evidence screened, assessed for eligibility, and included in the review, with reasons for exclusions at each stage, ideally using a flow diagram. | 3-4 |
| Characteristics of sources of evidence | 15 | For each source of evidence, present characteristics for which data were charted and provide the citations. | 27-36 |
| Critical appraisal within sources of evidence | 16 | If done, present data on critical appraisal of included sources of evidence (see item 12). | 6-8 |
| Results of individual sources of evidence | 17 | For each included source of evidence, present the relevant data that were charted that relate to the review questions and objectives. | 6-8 |
| Synthesis of results | 18 | Summarize and/or present the charting results as they relate to the review questions and objectives. | 6-8 |
| **DISCUSSION** | | | |
| Summary of evidence | 19 | Summarize the main results (including an overview of concepts, themes, and types of evidence available), link to the review questions and objectives, and consider the relevance to key groups. | 31-36 |
| Limitations | 20 | Discuss the limitations of the scoping review process. | 3-4; 10-11 |
| Conclusions | 21 | Provide a general interpretation of the results with respect to the review questions and objectives, as well as potential implications and/or next steps. | 6-12 |
| **FUNDING** | | | |
| Funding | 22 | Describe sources of funding for the included sources of evidence, as well as sources of funding for the scoping review. Describe the role of the funders of the scoping review. | 31-36 |

***Table S2:*** *Detailed overview of clinical trials and their characteristics. Listed are the publication and study ID from clinicaltrials.gov, the trial name (if available), the investigated purpose, the start year and duration, the study objective, the included patient characteristics, and the location.*

| **Reference** | **Trial Database ID** | **Trial name** | **Repurposing subject** | **Start date** | **Study duration** | **Intervention** | **Study population** | **Description of participants** | **Location** |
| --- | --- | --- | --- | --- | --- | --- | --- | --- | --- |
| Shah et al. 2017 | IRCT2015040721645N1 | - | actinic keratosis | 2013 | 2 years (2013 to 2014) | treatment | adults (over 18 years), both genders | actinic keratosis | Iran (Isfahan) |
| Tardif et al. 2021 | ACTRN12615000861550 | COPS | acute coronary syndrome | 2015 | 2 years and 10 months (December 2015 to September 2018) | treatment | adults (18-85 years), both genders | patients with acute coronary syndrome undergoing PCI or medical therapy | Australia |
| Vaziri et al. 2024 | ACTRN12615000485538 | COPE-PCI | acute coronary syndrome | 2017 | 2 years (December 2017 to 2019) | treatment | adults (18-85 years), both genders | patients with acute coronary syndrome undergoing PCI or medical therapy | Australia (Melbourne) |
| Shah et al. 2023 | NCT02140372 | - | anti-platelet effects | 2014 | 9 months (start May 2014, end January 2015) | treatment | adults (over 18 years), both genders | healthy adults | United States (New York City) |
| Rabbani et al. 2022 | ISRCTN32677164 | - | aphthous stomatitis | 2010 | 2 years and 8 months (July 2010 until April 2013) | treatment | adults (18-65 years), both genders | recurrent aphthous stomatitis | UK (London) |
| Gertner et al. 2024 | IRCT20141209020258N70 | - | cardiac surgery | 2013 | 2 years (2013 to 2014) | treatment | adults (over 18 years), both genders | open-heart surgery | Iran (Arak) |
| Lin et al. 2021 | IRCT201405308698N14 | - | cardiac surgery | 2014 | 9 months (November 2014 until July 2015) | treatment | no age limit, both genders | patients with open cardiac surgery | Iran (Teheran) |
| Demidowich et al. 2019 | ChiCTR2000040129 | - | cardiac surgery | 2020 | 2 years (November 30 2020 to 2022) | treatment | older adults (50-70 years), both genders | cardiovascular survery with cardiopulmonary bypass | China (Nanjing) |
| Shah et al. 2020 | IRCT20200328046886N6 |  | cardiac surgery | 2024 | 8 months (July 2024 to February 2025) | treatment | adults (18-80 years), both genders | coronary artery bypass graft surgery | Iran (Sari) |
| Shah et al. 2020 | ACTRN12610000293066 | LoDoCo | coronary artery disease | 2008 | 3 years and 10 months (July 2008 until May 2012) | prevention | older adults (35-85 years), both genders | patients with stable coronary artery disease | Australia |
| Tardif et al. 2019; Bouabdallaoui et al. 2020 | ACTRN12614000093684 | LoDoCo2 | coronary artery disease | 2016 | 2 years and 2 months (October 2016 until December 2018) | prevention | older adults (35-82 years), both genders | patients with stable coronary artery disease | Australia |
| Goldstein et al. 2022 | IRCT20200418047126N1 | - | COVID-19 | 2020 | 1 month (May to June 2020) | treatment | adults (18-75 years), both genders | COVID-19 infection in hospital setting | Iran (Ardabil) |
| Tabbalat et al. 2016 | IRCT20200408046990N2 | - | COVID-19 | 2020 | 5 months (May to September 2020) | treatment | adults (18-85 years), both genders | COVID-19 infection, mild to moderate, non-hospitalized | Iran (Mashhad) |
| **Reference** | **Trial Database ID** | **Trial name** | **Repurposing subject** | **Start date** | **Study duration** | **Intervention** | **Study population** | **Description of participants** | **Location** |
| Bessissow et al. 2018 | NCT04472611 | - | COVID-19 | 2020 | 1 year and 5 months (start October 30 2020, end March 30 2022) | treatment, comparison | adults (over 18 years), both genders | COVID-19 infection in hospital setting, non-critical ill | United States (Connecticut) |
| Shi et al. 2026 | NCT04355143 | - | COVID-19 | 2020 | 1 year and 3 months (start May 1 2020, end July 21 2021) | treatment | adults (over 18 years), both genders | COVID-19 infection in hospital setting, evidence of cardiac injury | United States (California, Florida) |
| Raeeskarami et al. 2022 | NCT04322682 | COLCORONA | COVID-19 | 2020 | 10 months (start March 23 2020, end January 21 2021) | treatment | adults (over 40 years), both males and females | COVID-19 infection in ambulant setting with high-risk characteristic | United States, Brazil, Canada, Greece, South Africa, Spain |
| Zhang et al. 2022 | NCT04392141; IRCT20150623022884N3 | - | COVID-19 | 2020 | 7 months (start April 1 2020; end November 1 2020) | treatment, comparison | adults and children (over 10 years), both men and women | COVID-19 infection in hospital setting | Iran (Kermanshah) |
| Amoli et al. 2015 | NCT04756128 | COLTREXONE | COVID-19 | 2021 | 10 months (start January 25 2021, end December 12 2021) | treatment | adults (over 18 years), both genders | COVID-19 infection in hospital setting, moderate- severe | United States (Minnesota) |
| Yousefzadeh et al. 2024 | IRCT20190804044429N5 | - | COVID-19 | 2021 | 5 months (February to May 2021) | treatment | adults (over 18 years), both genders | COVID-19 infection in hospital setting | Iran (Sari) |
| Mashayekhi et al. 2020 | IRCT20211017052786N1 | - | COVID-19 | 2022 | 5 months (January to May 2022) | treatment | adults (30-60 years), both genders | COVID-19 infection in hospital setting, intubated and connected to a ventilator | Iran (Qom) |
| Faghihi et al. 2016 | ChiCTR2500103522 | - | heart insufficiency (HFpEF) | 2024 | 1 year and 9 months (start January 2024, end September 2025) | treatment | adults (over 18 years), both genders | heart failure (HFpEF) and elevated inflammatory factors | China (Shijiazhuang) |
| Banifatemeh et al. 2025 | IRCT20111206008307N43 | - | heart insufficiency (HFrEF) | 2022 | 7 months (December 2022 to June 2023) | treatment | adults (18-80 years), both genders | heart failure (HFpEF) | Iran (Teheran) |
| Hejazi et al. 2024 | IRCT20111206008307N39 | - | heart insufficiency (HFrEF), myocardial infarction (STEMI) | 2022 | 1 year and 5 months (October 2022 until May 2023) | treatment | adults (over 18 years), both genders | HFrEF after recent STEMI | Iran (Teheran) |
| **Reference** | **Trial Database ID** | **Trial name** | **Repurposing subject** | **Start date** | **Study duration** | **Intervention** | **Study population** | **Description of participants** | **Location** |
| Taban-Sadeghi et al. 2025 | NCT01935700 | - | hepatocellular carcinoma, metastasis | 2013 | 6 years (start June 6 2013, end May 31 2019) | treatment | adults (20-90 years), both genders | HCC cancer, TNM stage 3b-4b | Taiwan (Kaohsiung City) |
| Salehzadeh et al. 2022 | NCT02153983 | - | metabolic disease; obesity | 2014 | 4 years and 2 months (start May 31 2014, end August 15 2018) | treatment | adults (over 18 years), both genders | obese adults without diabetes | United States (Maryland) |
| Kasiri et al. 2023 | ISRCTN12958502 | - | mycardial infarction (STEMI) | 2022 | 6 months (September 2022 to February 2023) | treatment | adults (30-65 years), both genders | STEMI | Indonesia |
| Farzaneh et al. 2026 | IRCT20210423051047N2 | - | mycardial infarction (STEMI) | 2022 | 1 year (May 2022 to 2023) | treatment | adults (over 20 years), both genders | patients with STEMI | Iran (Teheran) |
| Hosseini et al. 2022 | IRCT20231001059578N1 | - | myocardial infarction (STEMI) | 2022 | 2 years (2022 to 2023) | treatment | adults (18-80 years), both genders | patients with STEMI | Iran (Qom) |
| Khiali et al. 2024 | ACTRN12615001194550 | LoDoCo-MI | myocardial infarction | 2016 | 1 year and 6 months (February 2016 to July 2017) | treatment | adults (over 18 years), both genders | patients with acute myocardial infarction type 1 | Australia (Perth) |
| Akrami et al. 2021 | ACTRN12618000809235 | COCOMO-ACS | myocardial infarction (NSTEMI) | 2018 | 2 years and 9 months (December 2018 until March 2021) | treatment | adults (18-82 years), both genders | patients with acute non-STEMI myocardial infarction | Australia |
| Damanpak-Moghadam et al. 2026 | IRCT20120111008698N23 | PodCAST-PCI | myocardial infarction (STEMI), cardiac surgery | 2018 | 8 months (February to September 2018) | treatment | adults (over 18 years), both genders | acute STEMI, PCI procedure | Iran (Teheran) |
| Handari et al. 2025 | IRCT20201117049420N1 | - | myocardial infarction (STEMI, NSTEMI, unstable angina pectoris) | 2019 | 7 months (October 2019 until March 2020) | treatment | older adults (40-70 years), both genders | recent cardiovascular event | Iran (Shiraz) |
| Alsahaf et al. 2023 | NCT02594111 | COLCHICINE-PCI | percutaneous coronary intervention in coronary artery disease | 2013 | 8 years and 7 months (start May 30 2013, end December 31 2021) | treatment | adults (over 18 years), both genders | prior percutaneus coronary intervention due to systepcted ischemic heart disease or acute coronary syndrome | United States (New York City) |
| Vaidya et al. 2021 | NCT01709981 (substudy of NCT02594111) | COLCHICINE-PCI, substudy | percutaneous coronary intervention in coronary artery disease | 2015 | 4 years and 8 months (January 2015 until August 2019) | treatment | adults (over 18 years), both genders | prior percutaneus coronary intervention due to systepcted ischemic heart disease or acute coronary syndrome | United States (New York City) |
| Psalti et al. 2025 | ACTRN12619001231134 | - | percutaneous coronary intervention in coronary artery disease | 2015 | 2 years and 2 months (March 2015 to April 2017) | treatment | adults (over 18 years), both genders | acute coronary syndrome undergoing PCI | Australia (Sydney) |
| Hillis et al. 2019 | IRCT20191222045847N1 | - | PFAPA syndrome | 2019 | 2 years (2019 to 2021) | treatment | children (1 to 10 years), both genders | PFAFA syndrome | Iran (Teheran) |
| **Reference** | **Trial Database ID** | **Trial name** | **Repurposing subject** | **Start date** | **Study duration** | **Intervention** | **Study population** | **Description of participants** | **Location** |
| Tong et al. 2020 | NCT02551094 | COLCOT | reduction of cardiovascular events after myocardial infarction | 2015 | 4 years and 3 months (start April 12 2015, end July 17 2019) | prevention | adults (over 18 years), both genders | post myocardial infarction | Argentina, Chile, Colombia, France, Italy, Lebanon, Portugal, Spain, Tunisia, United Kingdom |
| Cole et al. 2022 | NCT03575572 | - | single-ventricle heart disease, post-operative | 2018 | 2 years and 7 months (August 2018 until January 2021) | treatment | children (20 months to 5 years), both females and males | fontan patients | United States (Michigan) |
| Nidorf et al. 2020 | NCT03021343; ISRCTN15501019 | END-AF | thoracic surgery, prevention of perioperative atrial fibrillation | 2012 | 2 years and 4months (start October 2012, end February 2015) | prevention | adults (over 18 years), both genders | open heart surgery (caused by non-defined conditions) | Jordan (Amman) |
| Nidorf et al. 2013 | NCT01985425 | COP-AF pilot | thoracic surgery, prevention of postoperative atrial fibrillation or atrial flutter | 2014 | 1 year and 6 months (start April 2014, end September 2015) | prevention | older adults (over 55 years), both genders | lung resection surgery | Canada |

***Table S3:*** *Aspects and indicators of a well-designed clinical trial methodology. Listed are the respective aspect, its importance, as well as good versus problematic properties and their consequences.*

| **Aspect** | **Importance** | **Good** | **Consequence** | **Problematic** | **Consequence** | **Reference** |
| --- | --- | --- | --- | --- | --- | --- |
| **Study status** | availability of information; delivery of the necessary sample size to ensure power and statistical significance; ability to answer the study question (effect, statistical significance) | completed | optimal situation to obtain high-quality, meaningful, and statistically significant results | interrupted, discontinued | depending on the reason for interruption, early termination must meet pre-established criteria and provide sufficient data to convince the majority of statisticians and clinicians of the validity of the conclusion; otherwise, positive findings may not be accepted or negative findings dismissed | Sica 2002; Williams et al. 2015 |
| **Number of participants** | influences statistical power, reliability, and generalizability; required number depends on purpose, methodology, and incidence | high number | more reliable results; higher likelihood of statistical significance and detecting differences between intervention and control groups | low number | lower statistical power; higher risk that results are unreliable or not statistically significant | Beck 2006; Kelly et al. 2010 |
| **Endpoint** | measures the trial effect; defines which aspects are used to assess intervention benefit | hard endpoint | reflects real-world effects; meaningful for physicians and patients; focuses on clinical relevance rather than laboratory markers | surrogate parameter | effect on a biomarker does not necessarily translate to improved outcomes; may overestimate benefit and create uncertainty even if the endpoint is reached | Campbell et al. 2018; Kemp and Prasad 2017 |
| **Placebo** | blinds whether patients receive the intervention or control; essential to reduce psychological effects and bias | placebo-controlled | ensures comparability between groups; prevents bias such as the “placebo effect” | no placebo | higher risk of bias due to psychological influence; placebo effect in intervention group vs. no effect in control may lead to overestimation of benefits or adverse events | Walach et al. 2005; Knezevic et al. 2025; Gupta and Verma 2013 |
| **Randomized** | ensures evaluation of efficacy in groups with similar characteristics; critical for assessing effect size | randomized | provides homogeneous and well-defined patient populations; minimizes selection bias | not randomized | increased risk of selection bias and result distortion | Sundaram et al. 2022; Lim and In 2019 |
| **Masked** | isolates the intervention effect from investigator influence; reduces bias; necessity depends on trial type and methodology | masked | ensures neutrality and avoids preconceptions | not masked, open label | higher risk of bias and distorted results; preconceived opinions may influence outcomes | Jones et al. 2022; Cristian et al. 2020 |
| **Sponsor** | provides financial resources for study execution but may introduce bias through vested interests | no industrial sponsor | independent; results less likely influenced by financial interests | industrial sponsor included | risk of biased interpretation in favour of sponsor; possible non-publication of unfavourable results | Lundh et al. 2012; Morrow et al. 2022 |
| **Safety evaluation** | determines risk-benefit profile; protects participants | assessment and reporting in publication | enables evaluation of risks vs. benefits and contextualization of potential benefits | limited reporting | restricts ability to assess risk-benefit; endangers patients if risk groups or severe adverse effects remain unknown; incomplete result interpretation | Martens and Logan 2024; Singh and Loke 2012; Yao et al. 2013 |
| **Limitations** | necessary for comprehensive interpretation, generalizability, and validity | transparent acknowledgement | allows accurate interpretation and assessment; improves understanding of informative value | not declared | omits restrictions; can cause misinterpretation or overestimation of findings | Kostis and Dobrzynski 2020; Rietdijk and Dräber 2024 |

***Table S4:*** *Literature findings and key results regarding efficacy, interpretation of repurposing potential, and safety. Additionally, limitations of the respective literature sources are provided.*

| **Reference** | **Repurposing subject** | **Key take-home messages** | **Interpretation of findings** | **Remarks on safety** | **Limitation** |
| --- | --- | --- | --- | --- | --- |
| Courtney et al. 2026 | atherosclerosis, calcific aortic valve stenosis | colchicine as inexpensive and anti-inflammatory medication; COLCOT trial reduction of cardiovascular events in adults with atherosclerotic disease; Co-Star showed reduction of new-onset atrial fibrillation; possible role of inflammatory processes in atherosclerosis | colchicine may be beneficial in atherosclerosis due to anti-inflammatory role in diseases considered an inflammatory component; significant benefit seen in trials | no consideration | subjective review without structured methodology; no consideration of safety |
| Alunno et al. 2025 | cardiovascular conditions | inflammatory processes have an important role in the development and progression of cardiovascular diseases; hyperuricemia being an independent CV risk factor; beneficial outcomes have been observed in clinical trials, but often also conflicting | hyperuricemia as risk factor for CV, therefore, urate-lowering therapies may be a beneficial prevention strategy, alongside with the anti-inflammatory effect of colchicine | no consideration | subjective review without structured methodology; no consideration of safety |
| Ding et al. 2026 | cardiovascular conditions | clinical trial data support efficacy in reduction of recurrence of pericarditis, long-term low-dose administration in atherosclerotic coronary artery disease reduces MACE; partly inconsistent results regarding an indication; benefits attributed to pleiotropic anti-inflammatory effects | proven benefit in some cardiovascular conditions while for others contradictory findings; benefit attributable to anti-inflammatory effects | despite naarow therapeutic inde, low-dose administration well-toleratied, gastrointestinal disturbances being the most common adverse effect; caution about drug interactions because CYP3A4 and P-glycoprotein involvement | narrative review without structured methodology |
| Leah et al. 2026 | cardiovascular conditions | cardiovascular disease with high burden and mortality worldwide; strategy of repurposing approved drugs in cardiovascular prevention; evaluation of pharmacoeconomic profiles and connection with clinical benefit and patient risks | colchicine considered as moderate cost-effectiveness ratio with morbidity benefit only | no serious adverse effects but common gastrointestinal effects have to be considered | narrative review without formal meta-analysis |
| Yoshida et al. 2021 | cardiovascular conditions | COLCOT trial for patients after MI found benefit of colchicine by risk reduction for primary composite cardiovascular endpoint (HR 0.77, CI 0.61-0.96), LoDoCO2 for patients with chronic CAD found benefit of colchicine by risk reduction for primary composite cardiovascular endpoint in the median of 29 months of follow-up (HR 0.69, CI 0.57-0.83); in trial with acute coronary syndromes did find a reduction in composite all-cause mortality but being non-significant (HR 0.65, CI 0.38-1.09) | colchicine is beneficial in high-risk patients in cardiovascular disease for secondary prevention, particular in comorbidity with gout | small bit significant increase in pneumonia in COLCOT trial (0.9% vs 0.4%); higher rate of all-cause death in two clinical trials | particular focus on benefit in gout patients; outdated - no inclusion of more recent trials |
| **Reference** | **Repurposing subject** | **Key take-home messages** | **Interpretation of findings** | **Remarks on safety** | **Limitation** |
| Moka et al. 2025 | cardiovascular conditions, geriatric population | anti-inflammatory properties may be effective in cardiovascular conditions like pericarditis and atherosclerotic cardiovascular disease; may be useful as adjunct to standard therapy | recommendation of drug repurposing of colchicine to achieve cardiovascular benefits, particularly in inflammatory-driven conditions; need for further evaluation to improve treatment efficacy of CV disorders | well-established safety profile, particular at low doses, fewer severe adverse effects compared to other anti-inflammatory drugs | subjective review without structured methodology; no comprehensive evaluation of available clinical trials and the emphasis of partly contradicting results or the finding that potential increase of non-cardiovascular-caused death |
| Misra et al. 2020 | cardiovascular conditions; COVID-19 | reduction of recurrent cardiovascular events, potential antifibrotic effects, but require further exploration; in disease model hints for improved survival and residual left ventricular function after MI, and reduction of uptake of 18-FDG in atherosclerotic plaques and reduced burden of atherosclerotic plaques and higher circulating HDL cholesterol; platelet aggregation reduced in vitro but not in vivo; no significant reduction in CRP levels after MI (LoDoCo-MI study); in STEMI no difference in CRP peak serum or significant outcomes for biochemical, clinical or imaging outcomes successfully met (COIN trial) | evidence of efficacy for prevention of CV events in special patient groups, further investigation needed in other patient groups such as primary prevention, metabolic syndrome and inflammatory arthritis; anti-fibrotic effects need further trials which are suitable powered; exploration of COVID-19 needed to be further evidenced; colchicine may hold promises to be repurposed for favourable modulation of cardiovascular risks and inflammatory mechanisms | rarely causing myopathies, cardiomyopathy, cytopenia and transaminitis; strong evidence needed to balance benefits and risk of adverse effects; greater incidence of pneumonia (0.9% vs 0.4% control, COLIN trial); hints for benefit in treatment of COVID 19 in regard to severity, progression and myocardial injury; often resulting in diarrhoea and other GI adverse effects | strong focus on adverse effects, but no mentioning of incidence or prevalence of common or severe side effects; mentioning of many experimental studies and clinical studies, but not all results resented and often no discussion of limitations and biases of trials and research |
| Moka et al. 2025 | cardiovascular disease (geriatric population) | colchicine with anti-inflammatory effects in cardiovascular conditions like pericarditis and coronary artery disease; reduction of major adverse CV events as add-on to standard therapies (COLCOT, LoDoCo2, risk reduction of 25-30% compared to placebo); promising results for atrial fibrillation and acute coronary syndrome, potentially decreasing associated complications; lowering of inflammatory surrogate parameters | potential of repurposing in elderly, need for more geriatric-specific trials | well-established safety profile, particular at low doses, fewer severe adverse effects compared to other anti-inflammatory drugs | limited data for geriatric specific, unverified for long-term safety, heterogeneity of included studies |
| Potere et al. 2024 | cardiovascular disease, secondary cardiovascular prevention | systemic inflammation represents major cardiovascular risk and prognostic factor and plays an important role and pathogenetic CVD development and progression | approvement of colchicine for prevention of secondary cardiovascular prevention for patients with coronary artery disease in the US; meta-analysis found colchicine treatment associated with 22% reduction in MACEs and reduced cardiovascular death, but no apparent total reduction in all-cause mortality; ongoing trials for other cardiovascular conditions | evidence of efficacy for secondary prevention of CV events, further but non-confirmed hints for further repurposing of cardiovascular diseases, additional research needed | comprehensive analysis of completed and ongoing trials on colchicine use, critical and balanced analysis with presentation of both significant and non-significant results, but no conclusive statement possible for other cardiovascular repurposing indications than secondary prevention in coronary artery disease |
| **Reference** | **Repurposing subject** | **Key take-home messages** | **Interpretation of findings** | **Remarks on safety** | **Limitation** |
| Alunno et al. 2025 | cardiovascular disease, secondary CV prevention in high-risk patients | significant long-term reduction for patients with risk factors for cardiovascular diseases with 0.5mg daily (trials LoDoCo and LoDoCo-2), mentioned in international guidelines as possible additional treatment | proven and significant effect of colchicine in the reduction of secondary cardiovascular events in high-risk patients, mentioned in guidelines; further investigation needed to implement broadly in clinical practice | - | referring to a low number of studies with regard to colchicine, no mentioning of studies with non-significant results or limitations of the presented studies |
| Dudam and Dekate 2020 | cardiovascular diseases | analysis of COLCOT trial; significant reduction in primary endpoint, including decreased rate of death from cardiovascular causes, resuscitated cardiac arrest, myocardial infarction, stroke and urgent hospitalisation for angina leading to coronary revascularisation | significantly lower risk of death or CV events; sufficient short-term evidence of benefits, looking forward future for long-term data | very well understood safety profile; slightly higher incidence of adverse events (16.0% colchicine vs 15.8% placebo), mainly diarrhoea (9.7% vs 8.9%) and nausea (1.8% vs 1.0%), pneumonia as a serious adverse event (0.9% vs 0.4%, p=0.03) | analysis of a small number of trials; |
| Gelosa et al. 2020 | cardiovascular diseases | potential benefits in wide range of conditions because of broad anti-inflammatory effect; experiments have shown that smooth muscle cell proliferation was suppressed and increasing cell apoptosis; recommendation by the European Society of Cardiology as first-line for acute and recurrent pericarditis and acute treatment of post-pericardiotomy syndrome | need of confirmation of benefit in STEMI underwent PCI and ACS by large RCTs | - | no reporting of studies with non-significant or contradicting results |
| Rodrígues-Zavala and Zazueta 2024 | cardiovascular diseases | new assigned indication: pericarditis (acute, recurrent), coronary artery disease, cardiovascular events and recent MI, ST-elevation, MI undergoing PCI, non-cardioembolic stroke; add-on fist-line treatment for pericarditis; benefits in patients with table CAD (LoDoCo2 trial), reduction of cardiovascular events in patients with T2D and recent MI (COLCOT trial); ongoing study evaluation CV in high-risk patients with type 2 diabetes (COLCOT-T2D trial), ad in ST-elevation MI undergoing PCI (CLEAR SYNERGY trial), and investigation of patients with non-cardioembolic stroke (CONVINCE trial) | successful repurposing for cardiovascular conditions; repurposing itself promising strategy but considers a wise development and conduction of powerful and suitable trials | - | brief examination of trials without in-depth-analysis of limitations and biases |
| Kao and Huang 2022 | cardiovascular diseases, immunomodulator | immunomodulator co-administered with coronary intervention; CRP used as a biomarker for inflammatory status; COLCOT study low-dose colchicine associated with significant reduction in subsequent ischaemic cardiovascular events; no effect on reduction of MACE within STEMI onset and PCI-related events (COLIN, COLCICINE-PCI) | inflammatory component important in cardiovascular disease; colchicine promising drug for inflammatory decrease; need for future clinical trials for generalisation of drugs | gastrointestinal adverse effects | comprehensive analysis of completed and ongoing studies, but no investigation of potential bias in studies, no conclusive statement of the role of colchicine or for repurposed studies |
| **Reference** | **Repurposing subject** | **Key take-home messages** | **Interpretation of findings** | **Remarks on safety** | **Limitation** |
| Chaffey et al. 2022 | cardiovascular inflammation | some studies found significant reduction (LoDoCo, LoDoCo 2, COLCOT for coronary artery disease), others did not find significant differences (COLCHICINE-PCI, COPS, COVERT-MI in PCI, MI, STEMI, acute coronary syndrome) | positive effect on outcome depends on investigated condition and study design; promising and cost-effective drug, but issues through adverse effects and narrow therapeutic index; further trials needed to confirm and validate effect | increased adverse GI symptoms in colchicine group, contraindicated for patients with chronic kidney disease, use correlated with adverse immunosuppressive effects, e.g. increased risk of pneumonia in COLCOT and increased rate of all-cause death in COPS, mainly due to increased incidence of sepsis, narrow therapeutic index presenting additional challenge in repurposing | No breakdown of the results/trials by condition and bias/limitation, no final evaluation carried out about assessment of colchicine as a repurposed drug |
| Mohammad et al. 2025 | clonal hematopoiesis of indeterminate potential (CHIP), clonal cytopenia of undetermined significance (CCUS) | in mice prevention of development of atherosclerosis in Tet2-mutation; in patients weakening of association between TET2 mutation and myocardial infarction risk; COLCOT trial substantially reduced VAF of TET2, showing potential to arrest clonal development in subgroup; no study has yet proven that a reduction in size/eradication of a clone is associated with improved outcomes; future studies needed to explore colchicine as prevention of CVD events in high-risk CHIP carriers | improvement of surrogate parameter is not similar to improved outcome; future studies needed to assess role and efficacy; recently COLCOT study found an effect but outcome of colchicine of CHIP has to be investigated | not specifically reviewed, but remark that repurposed drugs have an advantage to new developed drugs in terms of the prognosis and knowledge of adverse effects | hints available, but no conclusive statement possible |
| Ezzo and Etienne-Manneville 2025 | CNS cancer (glioma), neurodegenerative diseases | prolonged survival in mice, BBB penetration successful | colchicine effective in glioblastoma models/in vitro/in vivo | neurotoxicity is a concern for clinical development | experimental investigation, no clinical studies caried out/analysed |
| Bouabdallaoui and Tardif 2021 | coronary artery disease | demonstration of long-term use being associated with significant reduction in CV events in acute and chronic CAD (trails COLCOT and LoDoCo2); varying effectiveness in pericardial disease due to heterogenous trials | good option as an add-on for the long-term prevention of CV events in chronic CAD and after ACS; ongoing studies | overall good safety profile, possible gastrointestinal adverse effects (mainly nausea and diarrhea | focus on studies with significant and positive results, while not referring to studies in relation to cardiovascular disorders where no effect was found |
| Tien et al. 2021 | coronary artery disease (MI, PCI, mortality) | 10 studies included with 6398 patients; decreasing risk of CV events decreased significantly (OR 0.48, CI 0.28-0.79); protective effect on restenosis risk was found (OR 0.46, CI 0.23-0.92); no significant lowered risk of MI (OR 041, CI 0.16-1.08); no benefit found for all-cause mortality (OR 0.8, CI 0.56-1.15); high heterogeneity among the studies but more or less consistent findings | decreased risk of MI and restenosis after PCI or CAD patients, but no reduction in all-cause mortality; more trials needed; need to be aware of GI side effects | significantly higher risk of gastrointestinal events | outdated (access date April 28 2020); varying treatment durations and doses of colchicine; low event rates in some studies; clinical heterogenous |
| **Reference** | **Repurposing subject** | **Key take-home messages** | **Interpretation of findings** | **Remarks on safety** | **Limitation** |
| Montarello et al. 2022 | coronary artery disease, atherosclerosis | inflammation plays an important role cardiovascular diseases, contributes to worsening and CV events; targeting of inflammation important; colchicine therapy did significant reduce MACE, but did not reduce hs-CRP (COLCOT trial); benefit in prevention of cardiovascular disease in long-term use, reduction in MI, cardiac arrest and non-cardioembolic stroke (LoDoCo and LoDoCo2 trial); no statistical significant reduction after MI and no reduction of inflammatory biomarkers in sub-study (COLCOT trial); no significant difference in composite endpoint of all-cause mortality, ACS- ischemia-driven urgent revascularization and non-cardioembolic ischemic stroke, but in post hoc analysis for only cardiovascular death instead of total death, significant reduction was found (COPS trial) | emerging as an effective drug in patients with established CAD; anti-inflammatory drugs have the potential to prevent KCV events by slowing progression or reversion of subclinical atherosclerosis; further trials needed to identify beneficial targeted patient group and which biomarkers are appropriate to measure outcome | increased risk of pneumonia found in COLCOT trial; increased number of GI effects (15-20% of patients); increased higher total mortality for non-cardiovascular causes predominantly liked to sepsis provides cause for caution (LoDoCo2 trial) | no conclusive assessment of effects, no analysis whether results are significant or non-significant; no comprehensive analysis of bias and limitations of included trials and performed study |
| Liang et al. 2024 | COVID-19 | reduction of inflammatory lung injury and respiratory failure in experimental design with mice; trial with lower mortality and hospitalization rates in PCR-confirmed disease but none in total participants; effect by reducing cytokine storms, interfering with leukocyte activation; in vitro data supports efficacy; clinical trials suggest reduction of progression in mild-to-moderate cases, but without strong evidence for significant improving outcomes in severe cases | colchicine is promising candidate but efficacy is not finally proven | Abdominal pain, nausea, vomiting, diarrhoea, and hypovolemia, multiorgan failure | lack of critical examination, little discussion of opposing/non-significant/negative results, focus on studies and arguments that supports hypothesis |
| Danuma et al. 2023 | COVID-19 | colchicine use associated with mortality risk reduction of 32% (OR 0.68, CI 0.58-0.78, p=0.001), pooled estimates of mortality outcomes similar estimates (OR 0.73, CI 0.59-0.91); some studies with significant outcome, other non-significant; uncertain underlying mechanisms; varying results in studies of effect | performed pooled umbrella evaluation of meta-analyses found significant reduction in overall mortality in COVID-19 patients, uncertain outcome in vaccinated population since most trials were carried out in a time where vaccination was unavailable | studies differing in incidence and significance of adverse events | heterogenous pooled and biased analyses with varying outcomes and statistical significance; fast development and changes in standard treatment, large disconcordances |
| **Reference** | **Repurposing subject** | **Key take-home messages** | **Interpretation of findings** | **Remarks on safety** | **Limitation** |
| Vaz et al. 2023 | COVID-19 | clinical studies with positive results of clinical outcome compared with placebo, however, some carried out with a reduced number of patients causing confirmation bias in the results; large study COLCORONA with 21% risk reduction and NNT to prevent one primary end point event (death, hospitalization, mechanical ventilation) was 91; RECOVERY trial did not find a significant difference in 28-day mortality between colchicine and standard of care (RR 0.94, CI 0.83-1.07, p=0.32) and no significant difference in secondary endpoints, but a small but significant reduction in likelihood of progression to invasive mechanical ventilation or death (RR 0.86, CI 0.77-0.96, p=0.008); contradictory results partly explainable by dosage and study length | many studies performed; some with significant improvements but partly biased by small participant number, other found no difference, resulting in discrepant results; unsure effect of colchicine for COVD-19, potential effect, need or further studies of prolonged treatment; possibility that longer duration of treatment with reduced dose more effective in improving outcomes | favourable safety profile, no increase in adverse events compared to placebo in large COLCORONA trial | no reference to other biases in studies, like changes in standard of care and progress in COVID-19 treatment |
| Yasmin et al. 2022 | COVID-19 | colchicine led to significant reduction in COVID-19 severity (OR 0.41, CI 0.22-0.75, p=0.005) and CRP levels (WMD -19.99, CI -32.09 to -7.89, p=0.001); no significant difference in D-dimer levels, mechanical ventilation and all-cause mortality between colchicine and standard care; considered as a potential treatment for the "cytokine storm" caused by the hyperinflammation of the COVID disease | favourable role in COVID treatment; associated with a significant reduction of COVID-19 severity but not with a significant reduction in all-cause mortality; varying results and significance between studies; further trials needed to assess the efficacy and safety | - | limited number of included trials; varying duration of therapy and dosage |
| Ghaith et al. 2022 | COVID-19 | many studies with significant benefit of treatment with colchicine in COVID, but large trial RECOVERY no significant of reduction in mortality, hospital duration or disease progression | current evidence don’t support the use; studies are heterogenous and inconclusive; drug cannot be used in clinical practice or abandoned from research without studies with robust evidence; recently should not be used in clinic except for investigational purposes | narrow therapeutic range; high toxicity and drug interaction potential; dose adjustment necessary in patients with decreased liver and kidney function; typical adverse effects are not severe and including particularly gastrointestinal effects; overdose can result in severe adverse events and mortality | narrative review, no pooled analysis; conclusion is based on the results of the RECOVERY trial, while other studies with significant results were not considered stark |
| Brüssow 2021 | COVID-19 | large trials with colchicine showed a small or no survival benefit; small trial found less clinical deterioration than the control group (2% vs 14%, e.g. mechanical ventilation or death), significant results but cautious interpretation because of study size | insufficient evidence for the advantage of colchicine in COVID | safe; higher rate of adverse reactions than placebo, mostly diarrhoea | outdated (2021) |
| **Reference** | **Repurposing subject** | **Key take-home messages** | **Interpretation of findings** | **Remarks on safety** | **Limitation** |
| Lien et al. 2021 | COVID-19 | mortality risk significantly lower than control (OR 0.57, CI 0.38-0.87, p<0.01); no significant lower rate of mechanical ventilation (OR 0.67, CI 0.39-1.15, p<0.01); subgroup analysis did not result in statistically significant mortality reduction (OR 0.8, CI 0.44-1.46, p=0.22); differing findings between studies, some associated with better outcomes, other did not find any significant difference; heterogenous studies with significant publication bias | colchicine treatment significantly lower risk of mortality in COVID-19 patients, but not-significant in subgroup analysis; mechanism of action of colchicine on COVID remains unclear; further trials needed to confirm advantages of colchicine; no recommendation of routine colchicine treatment based on the systematic review and meta-analysis | mild and not significantly different to control (OR 2.03, CI 0.51-8.09) | quite outdated, comparison of heterogenous studies with biases |
| Elshafei et al. 2021 | COVID-19 | performed meta-analysis found significant lower mortality in the colchicine group (OR 0.35, CI 0.25-0.48, I^2 0%), with a subgroup analysis for hospitalized patients similar low and significant mortality reduction (OR 0.35, CI 0.25-0.5, I^2 0%); bias and limitations of the included studies, but no evidence of heterogeneity | potential benefit of colchicine in COVID 19; further trials needed | established safety profile; no increased or significant immunosuppressive status and predisposition to infections | limited number of included studies, all with comparable low participants size (9 studies, total 5522 patients), with majority of included patients (4488) not peer-reviewed; majority of studies observational, different dosages and duration of colchicine; early publication date with early stage in the treatment of COVID, speaking for improvements in the meantime for standard of care, likely reducing the effect and significance of colchicine treatment |
| Atzeni et al. 2021 | COVID-19 | anti-inflammatory action, reducing inflammatory response, particular in granulocytes and monocytes; one available study found reduction of death and hospitalization; need to wait for study results of further trails to evaluate effectiveness | no anti-inflammatory treatment has been shown to be clearly effective (status 2021); need to clarified effectiveness of colchicine in further trials and may be clinically useful | - | search performed in 2020 (January - October); low number of available studies (1), many ongoing trials |
| Yousefi et al. 2021 | COVID-19 | potential drug for anti-inflammatory response and cytokine storm suppression in COVID-19 patients; evidence of clinical improvement by reducing the length of hospitalization and dependency on supplemental oxygen therapy by trials | colchicine may be used for COVID-19 infections; considered as potentially candidate for clinical trials and further confirmatory studies | - | outdated (2020), lower number of included studies, no comprehensive analysis of studies but more of a broad overview of COVID-19 therapy options; no consideration of limitations and bias of referred trials |
| **Reference** | **Repurposing subject** | **Key take-home messages** | **Interpretation of findings** | **Remarks on safety** | **Limitation** |
| Hossen et al. 2020 | COVID-19 | mechanism of action could be the inhibition of virus entry, transport and replication by blocking microtubules polymerisation; activity against flaviviruses found but no precise data for COVID-19 patients; no recommendation for or against use by NIH; might be preventing cytokine stark and decrease COVID-19 severity | might be effective as a treatment option for COVID-19 but need more investigation for recommendation | common interactions with Abacavir, alogliptin, ampicillin, tianeptine, sulindac, ritonavir, vilanterol, zaleplon, and others; adverse effects include alopecia, diarrhoea, vomiting, leukopenia, granulocytopenia, thrombocytopenia, pancytopenia, aplastic anaemia, myopathy, elevated CPK, myotonia, muscle weakness, rhabdomyolysis; safety concerns include risk of GI perforation, renal impairment, hepatic impairment | outdated, no previous investigation of performed and ongoing trials, brief referring without presenting details or limitations of clinical trials; presents more an overview about characteristics instead of precise information and details (no measure values) |
| Louchet et al. 2020 | COVID-19, use in pregnancy | anti-inflammatory and immunomodulatory effects, may reduce cytokine storm and inflammatory cascade activation in severe COVID-19; to date has not been proved to be effective as a treatment or prevention of COVID-19 (2020); crosses the placental barrier; no increase in incidence of miscarriage, but increased risk for preterm deliveries under 36 weeks gestation (15% vs 5.9%, p<0.01) and lower median birthweight (3090g vs 3315g, p<0.01) | safe for pregnant women; potential candidate for COVID-19 treatment | increased risk for preterm deliveries and lower birthweight | investigation of side effects based on trial for Mediterranean fever and not patients with COVID; outdated; no analysis of bias and limitations of referred sources |
| Mathiesen et al. 2025 | diabetes type 1 | role of inflammatory processes in diabetes type 1; cardiovascular risks elevated, particularly for atherosclerotic cardiovascular diseases; evidence that colchicine has beneficial effect on CV and atherosclerosis progress; design of REC1TE trial for investigation of CV in diabetes type 1 patients | need of evaluation of role of colchicine in CV risk of diabetes type 1; design of clinical trial | not investigated yet | introduction of rationale and design of running trial without any results presented |
| Franceschin et al. 2024 | inflammatory skin conditions | anti-neutrophilic effects; mostly off-label uses; requirement of validation from robust clinical trials needed; conditions like autoimmune bullous disorders, sweet syndrome and pyoderma gangrenosum, pustular psoriasis, hidradenitis suppurative, behecet syndrome; effects include disruption of microtubule formation, reduces neutrophil migration and activity, decreases inflammatory cytokines | off-label use, decreasing inflammatory effects promising for dermatological conditions, requirement of research to establish efficacy and safety | considered as safe and effective, favorable safety profile, cautious use in patients with renal and liver dysfunction, well-tolerated at moderate doses, higher doses associated with significant increase in gastrointestinal adverse effects (diarrhea, nausea, vomiting), less common effects myelosuppression, myotoxicity, alopecia, hepatotoxicity | no comprehensive assessment of clinical studies with significant and non-significant results |
| Matzda Porges et al. 2026 | oncology | proposal that inflammatory processes are important in tumor initiation, enhancing angiogenesis and genomic instability; CANTOS trial demonstrated the reduction of IL-1 beta being associated with decrease in lung cancer, but other trials contradicted these findings | possible potential of colchicine by the reduction of inflammatory processes connected to tumor evolution; scarce clinical investigation | mild gastrointestinal symptoms, long-term use of low-dose colchicine considered well-tolerated and safe | subjective review without structured methodology; no evaluation of colchicine in malignant conditions in clinical trials |
| **Reference** | **Repurposing subject** | **Key take-home messages** | **Interpretation of findings** | **Remarks on safety** | **Limitation** |
| Dipasquale et al. 2026 | PFAFA, periodic fever syndrome in children | most common periodic fever syndrome in children; GCR agonists stop attacks but may shorted fever-free intervals and association with serious long-term adverse effects; reduction of attack frequency and prolonged attack-free intervals along with lowered GCR agonist use when continuous colchicine intake | colchicine as promising candidate for prophylaxis with reduction of GCR agonist need; further investigation necessary | common mild gastrointestinal adverse effects, discontinuations uncommon | review (systematic search); reliance of analysis on a few clinical trials and sparce evidence |
| Nidorf 2023 | secondary prevention of cardiovascular disease | LoDoCo pilot study with 532 patients, significant reduction of cardiac death and events in patients with chronic coronary disease; LoDoCo2 with 5522 patients, late intolerance in 3.4% and reduction of risk for adverse cardiovascular outcomes by 30%; COLCOT similar benefits; no reduction in LDC levels but reduction hs-CRP and IL-6 levels; colchicine included in guidelines in many countries (Europe, Canada, South America, Taiwan) | promising repurposing indication for colchicine | long-term use safe up to 2.mg daily; dose adjustments necessary for patients with decreased renal and liver function, contraindication in neutropenic patiens; drug interactions with P450 3A4 and P-glycoprotein inhibitors, occurrence of myalgia in LoDoCo2 trial, no evidence of increase in risk of death related to any specific cause by LoDo Co2 | lack o critical evaluation of disadvantages, limited discussion on adverse effects, lack of heterogeneity and subgroup analysis discussion, limited discussion of long-term safety, strong reliance on positive trials; no mentioning of trials with no significant effect |

***Table S5:*** *Dosage and effects of colchicine (Ghaith et al. 2022). Shown are the ranges in which colchicine dosage is considered subtherapeutic (no effect), therapeutic, toxic, or lethal. Additionally, an example dosage is provided for an average-weight person of 70 kg.*

| **Effect** | **Subtherapeutic** | **Therapeutic** | **Toxic** | **Lethal** |
| --- | --- | --- | --- | --- |
| **Concentration in mg/kg** | >0.015 | 0.015-0.03 | 0.5-0.8 | <0.8 |
| **Example dosage for average weighted person (70kg)** |  | 1-2mg | 35-56mg | 56mg |

***Table S6:*** *Overview of colchicine dosage in the analysed trials and the considered therapeutic range (thresholds see Table S5). Additionally, information on observed adverse events is provided. Notably, trials with potentially toxic colchicine concentrations show a higher rate and severity of adverse effects.*

| **Reference** | **Loading dosage and frequency of application** | **Maintenance dose** | **Accumulated dose per 24h** | **Interpretation effect by dosage**  **(Table S5)** | **Clinical trial results regarding adverse effects and safety** |
| --- | --- | --- | --- | --- | --- |
| Tardif et al. 2019 | - | 0.5mg once daily | 0.5mg | subtherapeutic | higher prevalence of pneumonia (0.9% colchicine vs. 0.4% placebo group, p=0.03); most adverse events were gastrointestinal, particularly diarrhoea |
| Vaziri et al. 2024 | - | 0.8mg one daily | 0.8mg | subtherapeutic | - |
| Tardif et al. 2021 | 0.5mg twice per day for the first 3 days | 0.5mg once daily | 1.0mg/ 0.5mg | subtherapeutic-therapeutic | similar number of adverse events (colchicine group 4.9% vs. placebo group 6.3%, p=0.051), pneumonia less frequent in the colchicine group (2.9% vs 4.1% in placebo, p=0.021); diarrhoea in 13.7% of colchicine group and 7.3% placebo group (p<0.0001) |
| Shah et al. 2023 | 0.6mg twice per day for the first 3 days | 0.6mg once daily | 1.2mg/ 0.6mg | subtherapeutic-therapeutic | in colchicine group higher but non-significant prevalence of diarrhoea (3.2% vs 0%, p=0.12) and myalgia (2.4% vs 0%, p=0.25), significant higher AST (6.0 vs 0.0 IU/L, p=0.0005) and ALT (17.0 vs 9.0 IU/L, p=0.01) from baseline |
| Bessissow et al. 2018 | - | 0.6mg twice per day | 1.2mg | therapeutic | low number of adverse events, similar rate in both groups |
| Demidowich et al. 2019 | - | 0.6mg twice per day | 1.2mg | therapeutic | no significant difference in adverse event rates |
| Gertner et al. 2024 | - | 0.6mg twice per day | 1.2mg | therapeutic | most common adverse effect was diarrhoea in colchicine groups prevalence of 12%); more likely to experience nausea/stomach pain/vomiting compared to standard of care alone; relation of mortality cases to study participation |
| Rabbani et al. 2022 | - | 0.6mg twice per day | 1.2mg | therapeutic | 4 patients terminated early due to adverse effects vs no patient in the control arm reported side effects |
| Shah et al. 2016 | 1.2 mg (once) | 0.6mg (once) | 1.8mg | therapeutic | - |
| Shah et al. 2020 (main study and sub study) | 1.2 mg (once) | 0.6mg (once) | 1.8mg | therapeutic | no difference in chest pain between groups (8.1%, p=0.45), gastrointestinal symptoms more frequently in the colchicine (9.3%) vs placebo (3.2%) group (p=0.001), other less frequently occurring adverse effects |
| Lin et al. 2021 | - | 1mg three times per day for 4 days, followed by 3 days break | 3.0mg/0.0mg (1.8mg average week cycle) | therapeutic - toxic | significantly higher incidence of biliary tract obstruction (p=0.0184), diarrhoea (p=0) |
| Tabbalat et al. 2016 | 2.0mg 12-24 hours prior surgery; 1.0mg 0-4 hours prior surgery | 0.5mg twice per day | 3.0mg/1.0mg | therapeutic - toxic | high prevalence of diarrhoea in colchicine group (24.6% vs 5.5% control, p<0.001), led to discontinuation in 23 patients of colchicine group (12.8%); anorexia in 11 colchicine and 4 control patients, non-significant (p=0.07) |
| Goldstein et al. 2022 | - | 0.6mg once daily | 0.6mg | therapeutic (children) | most common adverse effect was gastrointestinal; rate in historical controls vs. investigation group similar regarding nausea and vomiting |
| Akrami et al. 2021 | - | 0.5mg once daily | 0.5mg | subtherapeutic | significantly higher rate of gastrointestinal adverse effects in colchicine group, drop-out of two patients because of diarrhea |
| Alsahaf et al. 2023 | - | 0.5mg once daily | 0.5mg | subtherapeutic | significantly higher adverse effect rate in colchicine group, led to drp out of patients; reports of gastric pain, peripheral neuropathy and alopecia |
| **Reference** | **Loading dosage and frequency of application** | **Maintenance dose** | **Accumulated dose per 24h** | **Interpretation effect by dosage**  **(Table S5)** | **Clinical trial results regarding adverse effects and safety** |
| Amoli et al. 2015 | - | 1mg once daily | 1mg | therapeutic | no significant drug adverse effects |
| Banifatemeh et al. 2025 | 1 mg colchicine prior and  1 mg after PCI | - | 2mg | therapeutic | no information provided |
| Cole et al. 2022 | 1 mg colchicine followed by 0.5 mg 1 hour later | - | 1.5mg | therapeutic | few safety adverse events and similar in both groups |
| Damanpak-Moghadam et al. 2026 | - | 2mg once daily | 2mg | therapeutic | no information provided |
| Faghihi et al. 2016 | - | 1% gel (topical) twice daily | n.a. | n.a. | no severe adverse events |
| Farzaneh et al. 2026 | 1 mg colchicine twice at the first day | below 70kg: 0.5 mg; over 70 kg: 1 mg | 1.5-2mg/0.5-1mg | subtherapeutic-therapeutic | comparable rate of adverse effects, no significant difference |
| Handari et al. 2025 | 1 mg colchicine prior and  0.5 mg after PCI | 0.5mg once daily | 1.5mg/1mg | therapeutic | gastrointestinal events (particularly diarrhea), were common, but manageable and no serious adverse events |
| Hejazi et al. 2024 | - | 1mg once daily | 1mg | therapeutic | no severe adverse events |
| Hillis et al. 2019 | - | 0.5mg once daily | 0.5mg | subtherapeutic | well tolerated, no serious adverse effects |
| Hosseini et al. 2022 | 1mg (once) | 0.5mg once daily | 1.5mg/0.5mg | subtherapeutic-therapeutic | well tolerated, no serious adverse effects |
| Kasiri et al. 2023 | 2mg (once) | 1mg once daily | 2mg/1mg | therapeutic | no major adverse effects |
| Khiali et al. 2024 | - | 1mg once daily | 1mg | therapeutic | diarrhea and nausea were the major adverse effect; gastrointestinal adverse effects have to be taken in account in treatment decision |
| Mashayekhi et al. 2020 | 1mg twice a day for one day | below 70kg: 0.5 mg; over 70 kg: 1 mg | 2mg/0.5-1mg | subtherapeutic-therapeutic | no major adverse effects |
| Nidorf et al. 2013 | - | 0.5mg once daily | 0.5mg | subtherapeutic | no serious drug adverse effects |
| Nidorf et al. 2020 | - | 0.5mg once daily | 0.5mg | subtherapeutic | withdrawal of 32 patients (11%) because of intestinal intolerance |
| Pan et al. 2023 | - | 0.5mg once daily | 0.5mg | subtherapeutic | no serious adverse events associated with colchicine intake |
| Psaltis et al. 2025 | - | 0.5mg once daily | 0.5mg | subtherapeutic | increased rate of diarrhea (9.2% of participants) |
| Raeeskarami et al. 2022 | - | below 15kg: 0.25mg;  15-30kg: 0.5mg  over 30 kg: 1 mg | 0.25-1.0mg | therapeutic (children) | no higher incidence in coparison of placebo group |
| Salehzadeh et al. 2022 | - | 1mg once daily | 1mg | therapeutic | no serious adverse events |
| Shi et al. 2026 | - | 0.5mg once daily | 0.5mg | subtherapeutic | not provided |
| **Reference** | **Loading dosage and frequency of application** | **Maintenance dose** | **Accumulated dose per 24h** | **Interpretation effect by dosage**  **(Table S5)** | **Clinical trial results regarding adverse effects and safety** |
| Taban-Sadeghi et al. 2025 | - | 1mg once daily | 1mg | therapeutic | 4.1% incidence of adverse reactions; mild manifestations, no serious adverse effect; most common gastrointestinal effects (diarrhea, flatulence); transient renal function abnormalities with a decrease in eGFR |
| Tong et al. 2020 | 0.5 mg twice daily (first month) | 0.5mg once daily | 0.5mg | subtherapeutic | well tolerated, no serious adverse effects |
| Vaidya et al. 2021 | 1 mg colchicine prior and  0.5 mg after one hour | - | 1.5mg | therapeutic | no differences in rates of reported asverse effect in colchicine vs placebo |
| Yousefzadeh et al. 2024 | 2mg (once) | 0.5mg once daily | 0.5mg | subtherapeutic | no adverse events |
